# Cosmetic Preservative Potential and Chemical Composition of *Lafoensia replicata* Pohl. Leaves

**DOI:** 10.3390/plants13152011

**Published:** 2024-07-23

**Authors:** Débora Machado de Lima, Anna Lívia Oliveira Santos, Matheus Reis Santos de Melo, Denise Crispim Tavares, Carlos Henrique Gomes Martins, Raquel Maria Ferreira Sousa

**Affiliations:** 1 Chemistry Instituto, Federal University of Uberlândia, Av. João Naves de Ávila 2121, Uberlândia 38400-902, MG, Brazil; 2 Institute of Biomedical Sciences, Federal University of Uberlândia, Av. João Naves de Ávila 2121, Uberlândia 38400-902, MG, Brazil; 3 University of Franca, Av. Dr. Armando Salles Oliveira, 201, Franca 14404-600, SP, Brazil

**Keywords:** antioxidant, antimicrobial, cytotoxic, cosmetics, Lytraceae

## Abstract

The study evaluated the preservative potential of *Lafoensia replicata* Pohl. leaf extracts in cosmetics, highlighting their antioxidant, antimicrobial, and in vitro cytotoxic activities for ethanolic extract prepared by the maceration and tincture method. Total phenol content showed a higher phenol concentration in ethanolic extract and tinctures, and by LC-MS/MS-ESI-QTOF analysis, flavonoids, hydrolyzed tannins, and phenolic acids were identified. The ethanolic extract and tincture showed high antimicrobial activity against *Staphylococcus aureus*, *Pseudomonas aeruginosa*, and *Candida albicans* (MIC < 50 µg mL^−1^), high antioxidant activity (EC_50_ < 50 µg mL^−1^ in the DPPH method, and results > 450 µmol trolox equivalent in the ABTS and FRAP method), and low cytotoxicity in human keratinocytes (IC_50_ > 350 µg mL^−1^). The results suggest these extracts could be an alternative to synthetic preservatives in the cosmetic industry.

## 1. Introduction

The use of cosmetics (e.g., soaps, shampoos, skin creams, etc.) in people’s daily hygiene routine is widespread. The market for these products is constantly growing and is particularly important for the economy [1,2]. With the rise of social media, an increasing number of consumers search the internet for health and well-being information. Easy access to receipts has prompted some individuals to create cosmetics using natural ingredients. This process, commonly called DIY (do it yourself), may lead the general population to believe that these homemade cosmetics are safer than commercially produced ones, mainly when natural products are used [3].

Generally, cosmetics are susceptible to degradation, such as changes in the organoleptic characteristics (color, texture, and smell), fungal proliferation, pH variation, and viscosity. To prevent some of these unpleasant effects, chemical preservatives are added to inhibit the growth of microorganisms during the cosmetic’s shelf life. Esters of 4-hydroxybenzoic acid—known as methyl, ethyl, propyl, and butylparaben—are the most widely used preservatives in the cosmetics industry. They are chosen due to their ability to act against a broad spectrum of microorganisms (i.e., Gram-positive and Gram-negative bacteria and fungi). Moreover, they are easily soluble and have good sensitivity to pH variations. However, adverse effects, such as sensitization through contact and their ability to interfere with the endocrine system [4,5], might occur when parabens are employed. In addition, due to the rise of pro-ecological trends and the search for a sustainable lifestyle, cosmetics producers are increasingly looking for alternatives to replace synthetic preservatives with compounds of natural origin. 

Natural products have been used for medicinal purposes and skin care since ancient civilizations. Plant extracts are used due to their antioxidant capacity (natural preservative), pigmentation, and inhibition of microbial activity, which can also be beneficial in preventing various diseases [6]. Furthermore, several companies use plant extracts such as *Aloe vera*, *Persea americana*, *Bambusoideae*, and *Matricaria chamomilla* to formulate moisturizers, shampoos, soaps, conditioners, and hair care products. The compounds in these extracts need to present significant antimicrobial and antioxidant activities to act as preservatives. 

Cerrado is one of South America’s largest biomes, occupying around 23% of the Brazilian territory. It holds 12,829 native species and plays an important social role, as many populations survive on its natural resources and have traditional knowledge of its biodiversity [7]. The genus Lafoensia is among the species found in Cerrado, and some metabolites have already been isolated. However, despite its wide variety of species, few studies are related to the genus. Most studies are about *L. pacari*, used in folk medicine to treat gastric ulcers, scarring, tonics, back pain, and cancer, and with some compounds [8,9,10]. 

Another species that belongs to this genus is *L. replicata* Pohl., which is often confused with *L. pacari*, as they are visually very similar, differing in the crests less pronounced in *L. replicata*. There are no studies about the chemical composition or biological activity of *L. replicata* Pohl. Ethnopharmacological studies with the local population from eastern Maranhão (northeast region of Brazil) show the use of *L. replicata* in liver disease, inflammation, healing, curing, or relieving kidney problems, gastritis, high blood pressure, headaches, and stomach aches [11]. 

Previous studies have demonstrated that *L. replicata* contains tannins and a high concentration of total phenols, which may suggest potent antioxidant, anti-inflammatory, and healing properties [12,13]. As no previous study has been found for this plant, and with the growing demand for natural products to replace parabens, *Lafoensia replicata* emerges as a promising alternative for use in cosmetic formulations, where these activities are fundamental for effective preservative action.

This study evaluated the preservative potential of *L. replicata* leaf extract prepared by maceration and tincture through cytotoxicity, antioxidant, and antimicrobial activity tests. In addition, the chemical constituents of the extracts were obtained using mass spectrometry. 

## 2. Material and Methods

### 2.1. Plant Material

Leaves from *L. replicata* were collected on the Brazilian Federal Road BR-497 (18°59′20.3″ S 48°25′14.5″ W) in June 2021. Dr. Taciana B. Cavalcanti, from the Brazilian Agricultural Research Corporation (EMBRAPA), confirmed the plant’s identification. The voucher specimen was deposited in the Herbarium of the Federal University of Uberlândia (HUFU 82057) and the CEN Herbarium of the Embrapa Genetic Resources and Biotechnology (CEN 121433). This study was registered in the National System for the Management of Genetic Heritage and Associated Traditional Knowledge (SisGen) to access the plant material (AFB587D).

### 2.2. Extract Preparation

#### 2.2.1. Ethanolic Extracts

The leaves were dried in an incubator (BOD Nova Ética, São Paulo, Brazil, model 411/FPD 155 L) with air circulation at 35 °C. The dried powdered leaves were initially extracted with *n*-hexane by maceration at room temperature for 48 h. The *n*-hexane extract (HE) was filtered, and a rotary evaporator removed the remaining solvent at 40 °C under reduced pressure. The same procedure was performed sequentially with 98% ethanol, obtaining the ethanolic extract (EE). The dry extract was stored in a glass flask at −5 °C.

#### 2.2.2. Tincture 

The tincture of *L. replicata* leaves was obtained through the maceration process [14] with 98% ethanol. The dried and crushed plant material was placed in contact with a solvent for 30 days at room temperature and protected from light. Two types of extracts were prepared. One was filtered and removed using a rotary evaporator at reduced pressure at 40 °C to obtain a dried tincture (DT). The other one was filtered and stored at room temperature in an amber glass bottle to get the commercial tincture (CT).

### 2.3. Total Phenolic Content 

The total phenolic content was determined according to a methodology described by Quaresma et al. [15]. In a conical glass test tube was added 0.5 mL of a solution of the extracts/tinctures (250 µg mL^−1^, methanol), 2.5 mL of the Folin–Ciocalteu solution (10% m v^−1^, water) and 2.0 mL of sodium carbonate solution (7.5% m v^−1^, water). The reaction was kept at 50 °C for 5 min, and the absorbance was recorded at 760 nm using a UV–Vis spectrometer (Thermo Fisher Scientific, Waltham, MA, USA, model Genesys 10S UV–Vis). The obtained results were expressed in mg gallic acid equivalent (GAE) per gram of extracts/tinctures using a calibration curve of gallic acid. All the analyses were carried out in triplicate. 

### 2.4. Antioxidant Activity

#### 2.4.1. DPPH Radical Assay

The DPPH procedure was performed according to the method described by Quaresma et al. [15]. In a conical glass test tube protected from light was added 0.2 mL of the extracts/tinctures prepared in different concentrations (HE—50 to 333.3 µg mL^−1^, EE and DT—0.08 to 8.30 µg mL^−1^, and BHT—0.14 to 1.70 µg mL^−1^) and 2.8 mL of the 2,2-diphenyl-1-picrylhydrazyl (DPPH) reagent (140 mg mL^−1^). The mixture was allowed to rest for 1 h at room temperature, and the absorbance was measured at 517 nm (UV–Vis spectrometer, Thermo Scientific model Genesys 10S UV–Vis). Each experiment was performed in triplicate. The CE_50_ values were determined by the equation:

(1) $$\mathrm{AA}={DPPH}_{sequestered}\left(\%\right)=(\frac{{Abs}_{control}\left({Abs}_{sample}-{Abs}_{blank}\right)}{{Abs}_{control}})100$$

where Abs_control_ is the absorbance of the methanolic solution of the radical DPPH, Abs_sample_ is the absorbance of the mixture (DPPH + sample), and Abs_blank_ is the absorbance of the sample in methanol.

#### 2.4.2. Ferric Reducing Antioxidant Power Assay (FRAP)

The FRAP analysis was adapted as described by Malta and Liu [16]. Solutions of 10 mM of the 2,4,6-tris(2-pyridyl)-s-triazine (TPTZ) dissolved in 40 mM HCl, 20 mM of ferric chloride dissolved in water, 0.3 M of the acetate buffer (pH 3.6), 1600 μM of Trolox in methanol (and standard curve between 25 and 480 μM), and 50 μM of extracts/tinctures in methanol were prepared separately. The FRAP reagent solution was prepared by mixing 100 mL of acetate buffer, 10 mL of TPTZ, and 10 mL of ferric chloride. The mixture was kept at 37 °C for 30 min. 0.15 mL of the Trolox/extracts/tinctures and 2.85 mL of the FRAP solution (previously prepared) were added in a conical glass test tube protected from light. The mixture was kept for 10 min, protected from light, and then the absorbance was measured at 593 nm (UV–vis spectrometer, Thermo Fisher Scientific model Genesys 10S UV–Vis). The results were expressed in µmol Trolox equivalent (TE) per gram of extracts/tinctures using a calibration curve of Trolox.

#### 2.4.3. ABTS

The ABTS evaluation was adapted as described in Malta and Liu [16]. Solutions of 7.4 mM ABTS, 2.6 mM of potassium persulfate, 25 to 600 mM of Trolox, and 50 μM of extracts/tinctures were prepared. The reaction mixture was obtained by mixing equal volumes of ABTS and potassium sulfate solutions and then kept in a dark environment for 12 h at room temperature. Subsequently, 1 mL of the reaction mixture was added to 60 mL of methanol to achieve an absorbance of 1.10 ± 0.02 A.U. at 734 nm. Fresh solutions were produced for each analysis. In a conical glass test tube protected from light, 0.15 mL of extract (HE, EE, and tincture) and 2.85 mL of the reaction mixture were added. The mixture was kept for 2 h, protected from light, and then the absorbance was measured at 734 nm (UV–Vis spectrometer, Thermo Fischer Scientific model Genesys 10S UV–Vis). The results were expressed in µmol of Trolox per gram of extract.

### 2.5. Determination of the Minimum Inhibitory Concentration Using the Microdilution Method

The antimicrobial activity was determined using the broth microdilution method according to the Clinical and Laboratory Standards Institute for Bacteria [17,18]. The microorganisms evaluated were from the American Type Culture Collection (ATCC): *Escherichia coli* (ATCC 8739), *Staphylococcus aureus* (ATCC 6538), *Pseudomonas aeruginosas* (ATCC 9027), and *Candida albicans* (ATCC 10231). The extracts/tinctures concentrations evaluated were 0.39 to 8000 µg mL^−1^ in dimethylsulfoxide (DMSO; Sigma-Aldrich, St. Louis, MO, USA; 5%, *v*/*v*). The positive control antibiotics analyzed were gentamicin for strains of *E. coli*, *S. aureus*, and *P. aeruginosa* at concentrations of 0.0115 to 5.9 µg mL^−1^ and amphotericin B for strains of *C. albicans* at concentrations of 0.031 to 16 µg mL^−1^. For bacteria, the inoculum in Mueller-Honton broth was adjusted in a spectrophotometer to give a cell concentration of 5 × 10^5^ colony-forming units per mL (CFU/mL) in the 96-well microplates.

For yeast, inoculum suspension was prepared in RPMI 1640 broth at 37 °C for 24 h and diluted to 1.2 × 10^3^ UFC mL^−1^. In the 96-well microplates, one inoculated well was included to control broth adequacy for microorganism growth, and one non-inoculated well free of antimicrobial agents was also used to ensure medium sterility. The microplates were incubated at 37 °C for 24 h. After that, 30 μL of resazurin (Sigma-Aldrich) at a concentration of 0.02% was added to each well. Resazurin is a redox probe that allows immediate observation of microbial growth. Blue and red represent the absence and presence of microbial growth, respectively [19]. The MIC is the lowest concentration that inhibits the growth of microorganisms. 

### 2.6. Cytotoxicity Assessment

According to Riss et al. [20], cytotoxicity assessment was performed using the resazurin colorimetric assay. The human non-tumor keratinocyte cell line (HaCat) was used in the cytotoxic analysis. Cells were cultured in Dulbecco’s modified Eagle medium (DMEM, Sigma-Aldrich) supplemented with 10% fetal bovine serum (Cultilab, Campinas, São Paulo, Brazil), antibiotics (0.01 mg mL^−1^ streptomycin and 0.005 mg mL^−1^ penicillin; Sigma-Aldrich), and 2.38 mg mL^−1^ Hepes (Sigma-Aldrich), at 36.5 °C with 5% CO_2_. The extracts/tinctures were dissolved in DMSO (Sigma-Aldrich; 1%) at concentrations ranging from 39 to 5000 µg mL^−1^. Therefore, 1 × 10^4^ HaCat cells were seeded in a 96-well plate. Negative (DMSO 1%) and positive (DMSO 25%) control cultures were included. After 24 h of treatment, the culture medium was removed, and the cells were washed with PBS (phosphate-buffered saline solution) to remove the treatments and exposed to 80 μL of Ham’s Nutrient Mixture F10 culture medium (HAM-F10) without phenol red (Sigma-Aldrich). Then, 20 μL of resazurin (0.15 mg mL^−1^) was added to each well. The 96-well plate was incubated at 36.5 °C for 4 h. The absorbance of the samples was determined using a multiplate reader (Biochrom, Cambridge, UK, model ELISA—Asys—UVM 340/MikroWin 2000) at a wavelength of 570 nm and a reference length of 600 nm. The experiments were performed in triplicate. A non-linear regression analysis was performed using the GraphPad Prism program to calculate the sample concentration that inhibits 50% of cell viability (IC_50_).

### 2.7. Analysis of the Extracts by Liquid Chromatography Coupled with Mass Spectrometry (LC-ESI-MS/MS)

The analyses were performed on the Maxis Impact mass spectrometer (Bruker) with an ESI-Q-TOF configuration and coupled to Nexera high-performance liquid chromatography (Shimadzu, Kyoto, Japan). The internal calibration of ESI-Q-TOF was performed with a solution of 100 µM sodium formate in water/acetonitrile (1:1) and data-dependent acquisition mode (DDA/AutoMS) with isolation/fragmentation of three precursors per cycle. The flow rate of the ESI-Q-TOF nebulizer gas was 8.0 L min^−1^ and all the data were acquired in negative modes. The extract solution was prepared at 2 mg mL^−1^ in methanol. The LC separation was performed with an Ascentis^®^ C18 column (150 mm × 1 mm × 3 µm, Ascentis, Supelco). The mobile phase A has 0.1% formic acid, and the mobile phase B has methanol. 10 µL of sample was injected at a flow rate of 0.075 mL min^−1^_,_ and the separation was performed using the following gradient: 5–75% B (1 to 5 min); 75–100% B (5–15 min); 100% B (15–24 min); 100–5% B (24–25.2 min); 5% B (25.2–32 min).

### 2.8. Preparation of Topical Moisturizing Cream and Evaluation of the Preservative Activity of the L. replicata

Four topical moisturizing creams were prepared according to the Brazilian Resolution [21] in collaboration with D’brisse^®^ company (Minas Gerais, Brazil). The cream base contains cetearyl alcohol, polysorbate 60, glycerin, PEG 100 stearate, rosehip, and water. The extracts/tinctures were dissolved in glycerin following their incorporation into the base cream until homogenized. The cream composition of the four formulations contained 5% (mass/mass) of glycerin and 0.4% (mass/mass) of EE, dried tincture, commercial tincture, or methylparaben.

### 2.9. Statistical Analysis

The Analysis of Variance (ANOVA) method was used to evaluate the results obtained in the analyses to determine the total phenol content and antioxidant activity. Those results with a significance level of less than 5% (*p* < 0.05) were considered statistically different. Tukey’s test was used to determine significant differences between the means. The analyses were carried out using SigmaPlot 11.0.

## 3. Results and Discussion

### 3.1. Determination of Total Phenolic Content and Antioxidant Activity 

The total phenolic content (TP) was determined using an analytical curve relating the absorbance generated by the reaction with the Folin–Ciocalteu reagent to the concentration of gallic acid. A linear equation was obtained (y = 0.0147x + 0.0554) with a coefficient of determination (r^2^) of 0.9904. The TP content was expressed in milligrams of gallic acid equivalent per gram of extract (mg EAG g^−1^). If a high value of phenolic compounds is found in an extract, it may partly explain its antioxidant activity. The results obtained for TP content and the antioxidant activities (DPPH, FRAP, and ABTS methods) are shown in Table 1.

TP contents in EE and DT were 230 and 253.7 mg GAE g^−1^. Table 1 shows the variation in the reducing power of the extracts using the FRAP methodology and the variation in radical scavenging (DPPH and ABTS). Both extracts showed high values obtained by both analyzed methods, which were associated with the high total phenol content.

The DPPH analysis revealed that these samples showed CE_50_ value very close to the BHT (2.58 ± 0.01 µg mL^−1^), a synthetic antioxidant used as a positive control. The tincture and ethanolic extract indicate promising results for the *L. replicata* plant.

Extraction with hexane revealed a low TP content in its composition. This difference can be explained by the difference in polarity between hexane and the polar properties of the ethanol used in the other extraction process. The hexane extract’s low number of phenolic compounds results in low antioxidant activity.

It has been reported that phenolic compounds are responsible for antioxidant activity through several potential pathways. The main one is probably through free radical scavenging, in which phenolic molecules can break down the free radical chain reaction [22]. Phenolic acids, flavonoids, coumarins, and tannins are compounds found in the species that may be directly related to this antioxidant activity [23].

### 3.2. Antimicrobial Activity and Cytotoxicity Assessment

The Brazilian resolution [24] establishes parameters for the microbiological control of personal hygiene products, cosmetics, and perfumes. The microorganisms *S. aureus*, *P. aeruginosa* and fecal coliforms were evaluated because they are likely found in cosmetics. The minimum inhibitory concentration of each extract was assessed separately for each microorganism, considering its possible use as a preservative in creams. Table 2 shows the results of the antimicrobial activity.

All the ethanolic extracts and tinctures of the plant inhibited the growth of the evaluated microorganisms, except for *E. coli*, with MIC values between 25 and 100 µg mL^−1^. The tincture was the most active against *S. aureus* and *P. aerugionosas*, with MICs of 25 µg mL^−1^ and 50 µg mL^−1^, respectively. For the fungus *C. albicans*, all the extracts except the hexane extract presented a MIC < 100 µg mL^−1^; moreover, low values (3.12 µg mL^−1^) were obtained, showing good antifungal activity. 

These results are relevant and promising since the ethanolic extract and tincture showed MIC values lower than 100 µg mL^−1^ for three of the microorganisms evaluated [25], as well as showing better results when compared to the *L. pacari* species (the ethanolic extracts of the leaves and stem showed a MIC of 312.5 and 625 µg mL^−1^ against *S. aureus*, respectively) [26]. Another study showed that the hydroalcoholic extract of *L. pacari* leaves had a MIC of 250 µg mL^−1^ for *P. aeruginosa* [27].

Cytotoxicity was measured using the non-tumor human keratinocyte cell line (HaCat), with the results expressed as the concentration that inhibits 50% of cell viability (IC_50_). Table 3 shows the results obtained and the selectivity index calculated.

The selectivity index (SI) was determined to obtain a relationship between the cytotoxic concentration and the antimicrobial activity, using Equation (2):

(2) $$\mathrm{SI}=\log\;(\frac{IC50}{MIC})$$

The SI of the extracts showed that for bacteria with a MIC of less than 50 μg mL^−1^, the extracts had low toxicity (SI > 0).

### 3.3. Analysis of the Effectiveness of the Preservative System of the Creams—“The Challenge Test”

Motivated by the promising results of total phenol content and antimicrobial and antioxidant activity for the ethanolic extract and tincture of *L. replicata*, the next step was to evaluate its use as a preservative in cosmetics. The Brazilian resolution [28] establishes the substances permitted for personal care products, cosmetics, and perfumes. Based on this resolution, a moisturizing cream was made with 0.4% methylparaben, one of the most widely used preservatives in the cosmetics industry.

The challenge test is used during a product’s development to determine the preservative’s effectiveness and stability over time. It is carried out by inoculating a known quantity of microorganisms (bacteria and fungi) protected from light and incubating for 28 days. The results are shown in Table 4.

Results in Table 4 show that all the creams presented a progressive reduction in the microbial load over time. From the seventh day onwards, there was a reduction in viable bacteria from the initial count, followed by a continuous decrease until the end of the test. This profile also occurred for the fungus *C. albicans*. For the cream produced with methylparaben, by the seventh day, there were no longer any microorganisms in the product. For the creams made with the tincture and ethanolic extract of the leaves of *L. replicata*, microorganisms were reduced substantially after seven days, and after 21 days, no more microorganisms were found. 

Two creams were made using the tincture. One used 2% commercial tincture (CT), and the other used 0.4% dried tincture (DT). For the cream using 2% CT, 21 days were needed for complete inhibition of the microorganisms. In comparison, for the cream using 0.4% DT, there was no more proliferation of bacteria and fungi after 14 days. This can be explained by the fact that 2% CT in 350 g of cream corresponds to a concentration of approximately 0.3% DT. 

For the cream to which no preservative system was added, there was no reduction in the number of microorganisms in the cream. Although the tincture and ethanolic extract of the plant’s leaves needed more days to inhibit microbial growth when compared to methylparaben, both are within the legal limit for use as preservatives (total inhibition of microbial growth for up to 28 days) [17].

Therefore, the extracts obtained from this species should be considered a promising natural source for other activities.

### 3.4. Chemical Composition

**Figure 1:**
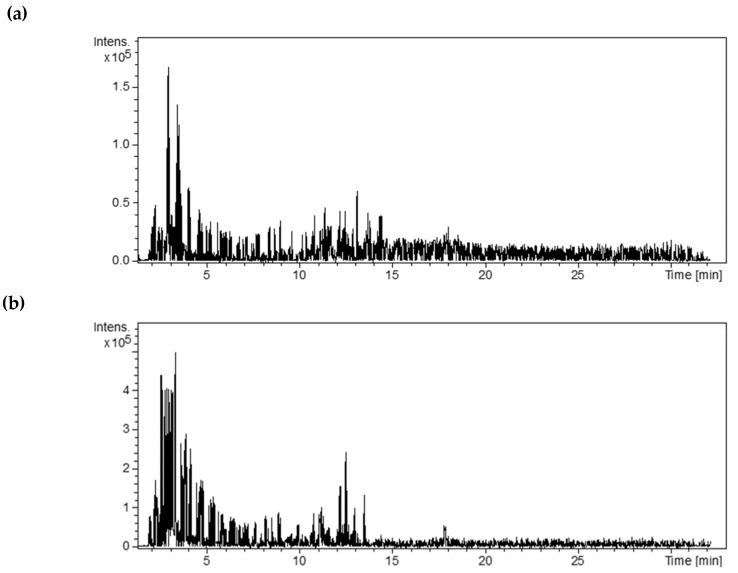
TIC chromatograms for (**a**) extracts and (**b**) tincture from leaves of the *L. replicata.*

The ethanolic extract and tincture showed better antioxidant and antimicrobial activity when compared to the hexane extract. For this reason, high-performance liquid chromatography coupled with mass spectrometry (HPLC-MS) with an electrospray ionization (ESI) system was used to obtain the chemical composition of the ethanolic extract and tincture. The analyses were carried out in negative mode. By analyzing the TIC chromatograms in Figure 1 and comparing the values of the *m*/*z* peaks obtained in the mass spectrum with a database of *m*/*z* values, it was possible to identify the composition of the main substances, as shown in Table 5.

EE and DT samples showed a predominance of tannins, flavonoids, and phenolic acids. Ellagitannins are hydrolyzable tannins that have attracted much attention because they have several beneficial properties for human health, including reducing the risk of diabetes, anticancer, and antioxidant activities [41].

Some studies have confirmed the biological activities of these compounds, such as antimicrobial, antioxidant, anti-inflammatory, and antimalarial activities [22,42]. Another compound belonging to the ellagitannin class that was also identified was pedunculagin (compound **3**), with a molecular ion at *m*/*z* 783, which produced a fragment ion at *m*/*z* 481, which corresponds to HHDP-glucose (compound **2**).

It is possible to note the many phenolic compounds identified by the Folin–Ciocalteu method, including acids, flavonoids, and tannins, which are possibly responsible for the extracts with high antioxidant and antimicrobial activity. Compounds **4**, **13**, **20**, **23** and **25** were found only in the DT sample. This is probably due to the longer extraction time.

## 4. Conclusions

The ethanolic, hexane, and tincture extracts of *L. replicata* were evaluated for the first time regarding chemical composition and biological activities. It is possible to correlate the presence of these compounds with the high content of total phenols and the antioxidant and antimicrobial activities of the extracts. LC-ESI-MS/MS enabled the identification of tannins, flavonoids, and phenolic acids in the most active extracts.

By analyzing the content of total phenols, it was possible to verify that both the ethanolic extract and the tincture showed a large number of phenolic compounds, high antioxidant activity (CE_50_ < 50 µg mL^−1^) for the DPPH method, values greater than 350 µmol ET g^−1^ for the FRAP and ABTS methods, and high antimicrobial activity (CIM < 50 µg mL^−1^). On the other hand, the hexane extract showed a low PT content and, consequently, low antioxidant and antimicrobial activity.

When comparing the creams prepared with extracts from *L. replicata* leaves and the cream prepared with methylparaben, it was observed that the extracts have the same ability to be used as preservatives, as they were able to reduce viable bacteria and fungi by at least 99.9% of the initial count. This statement also correlates to the activities found in this study. One can then consider the extracts obtained from *L. replicata* as a promising natural source for the study of other activities as well as a possible ally for the production of cosmetics.

## Figures and Tables

**Table 1:** Total phenol content and antioxidant activities by DPPH, FRAP, and ABTS methods of the extracts from leaves of the *L. replicata*.

Sample	Total Phenol Content (mg GAE g^−1^)	Antioxidant Activity		
		DPPH CE_50_ (µg mL^−1^)	FRAP (µmol TE g^−1^)	ABTS (µmol TE g^−1^)
HE	43.4 ± 1.8 ^a^	>200	84.67 ± 3.47 ^a^	111.3 ± 2.7 ^a^
EE	253.7 ± 2.6 ^b^	4.24 ± 0.16 ^a^	473.79 ± 6.82 ^b^	473.79 ± 46.3 ^b^
DT	230.2 ± 2.1 ^c^	3.50 ± 0.06 ^b^	681.16 ± 5.23 ^c^	479.7 ± 12.0 ^c^

Note: HE: hexane extract; EE: ethanolic extract; DT: dried tincture. Analyses with the same letter showed no significant difference between the means by Tukey’s test at 5% for the same test. A *p*-value < 0.01 was obtained for all correlations with different means.

**Table 2:** Minimum inhibitory concentrations (MIC) of the extracts from leaves of the *L. replicata*.

Microorganisms	Minimum Inhibitory Concentration (MIC)—µg mL^−1^				
	Extracts			Antibiotics	
	HE	EE	DT	Gentamicin	Anthofericin B
*S. aureus*	>800	50	25	0.36	-
*E. coli*	>800	>800	>800	0.36	-
*P. aeruginosas*	>800	100	50	0.36	-
*C. albicans*	400	3.12	3.12	-	0.5

Note: HE: hexane extract; EE: ethanolic extract; DT: dried tincture.

**Table 3:** Cytotoxicity assessment of the extracts from leaves of the *L. replicata*.

Samples	IC_50_ (µg mL^−1^)	Selectivity Index			
		*S. aureus*	*E. coli*	*P. aeruginosas*	*C. albicans*
EE	397.23 ± 1.70	0.90	−0.30	0.60	2.10
DT	396.87 ± 20.00	1.20	−0.30	0.90	2.10

Note: EE: ethanolic extract; DT: dried tincture; IC_50_: the concentration that inhibits 50% of the viability of human keratinocytes.

**Table 4:** Challenge test for the creams without any preservative (control) and with methylparaben and extracts from leaves of *L. replicata* to evaluate stability for 0, 7, 14, 21, and 28 days.

Preservatives	Microorganisms	0 Days log/CFU	7 Days log/CFU	14 Days log/CFU	21 Days log/CFU	28 Days log/CFU
-	*A. brasiliensis*	5.70	5.65	5.63	5.69	5.72
	*C. albicans*	6.91	6.74	6.74	6.88	6.84
	*E. coli*	7.20	7.11	7.31	7.20	7.12
	*P. aeruginosa*	6.18	6.14	6.14	6.21	6.26
	*S. aureus*	6.26	6.35	6.35	6.25	6.43
Methylparaben	*A. brasiliensis*	5.00	<1.00	<1.00	<1.00	<1.00
	*C. albicans*	6.08	<1.00	<1.00	<1.00	<1.00
	*E. coli*	6.14	<1.00	<1.00	<1.00	<1.00
	*P. aeruginosa*	6.15	<1.00	<1.00	<1.00	<1.00
	*S. aureus*	6.36	<1.00	<1.00	<1.00	<1.00
CT	*A. brasiliensis*	5.86	1.24	1.24	<1.00	<1.00
	*C. albicans*	6.43	1.70	1.70	<1.00	<1.00
	*E. coli*	6.60	3.21	2.30	<1.00	<1.00
	*P. aeruginosa*	6.49	3.18	3.18	<1.00	<1.00
	*S. aureus*	6.91	<1.00	<1.00	<1.00	<1.00
DT	*A. brasiliensis*	5.78	1.74	<1.00	<1.00	<1.00
	*C. albicans*	6.33	2.01	<1.00	<1.00	<1.00
	*E. coli*	6.46	2.12	<1.00	<1.00	<1.00
	*P. aeruginosa*	6.89	2.34	<1.00	<1.00	<1.00
	*S. aureus*	6.47	2.14	<1.00	<1.00	<1.00
EE	*A. brasiliensis*	5.63	<1.00	<1.00	<1.00	<1.00
	*C. albicans*	6.89	1.00	<1.00	<1.00	<1.00
	*E. coli*	6.89	2.31	<1.00	<1.00	<1.00
	*P. aeruginosa*	6.88	2.90	2.04	<1.00	<1.00
	*S. aureus*	6.71	1.30	<1.00	<1.00	<1.00

Note: CT: commercial tincture; DT: dried tincture; EE: ethanolic extract.

**Table 5:** Proposed identification of compounds in the extracts and tincture from leaves of the *L. replicata*.

	R_t_ (min)	Compound	Molecular Formula	*m*/*z* exp. [M − H]^−^	*m*/*z* Calculated [M − H]^−^	Error (ppm)	Fragments MS^2^	Extracts	Refs.
1	1.967	Sorbitol	C_6_H_13_O_6_^−^	181.0724	181.0718	3.31	163	EE; DT	[29,30]
2	1.967	HHDP—glucose	C_20_H_17_O_14_^−^	481.0635	481.0622	2.70	421, 301, 275	EE; DT	[31,32]
3	2.094	Pedunculagin isomer I (di-HHDP—glucose)	C_34_H_23_O_22_^−^	783.0685	783.0686	−0.12	481, 301, 291, 275, 249, 145	EE; DT	[31,32]
4 *	2.097	Pedunculagin isomer I (di-HHDP—glucose)	C_34_H_23_O_22_^−^	391.0303	391.0307	−1.02	301, 291, 275, 145	DT	[31,32]
5	2.178	Sucrose + formic acid	C_12_H_21_O_11_^−^	387.1166	387.1144	5.68	341, 179, 161, 143	EE; DT	[30]
6	2.220	Punicalin	C_34_H_21_O_22_^−^	781.0530	781.0530	0.00	765, 721, 601, 575, 481, 393, 301, 299, 273	EE; DT	[31,32]
7	2.473	HHDP galloyl glucose	C_27_H_21_O_18_^−^	633.0733	633.0733	0.00	463, 275, 301, 249, 169	EE; DT	[31,32]
8 *	2.490	Punicalagin isomers	C_48_H_27_O_30_^−^	541.0269	541.0260	1.66	601, 531, 402, 301, 124	EE; DT	[32]
9	2.515	Galloyl glucose	C_13_H_15_O_10_^−^	331.0686	331.0671	4.53	304, 170, 169, 139, 125	EE; DT	[31]
10	2.541	Trisgaloyl—HHDP glucose	C_41_H_27_O_27_^−^	951.0784	951.0740	4.62	907, 605, 425, 341, 301, 275	EE	[31]
11	2.566	Galloyl punicalin	C_41_H_25_O_26_^−^	933.0627	933.0640	−1.39	631, 451, 425, 301	EE; DT	[31]
12	2.617	Gallic acid	C_7_H_5_O_5_^−^	169.0139	169.0142	−1.77	125	EE; DT	[31]
13	2.687	Flavogalonic acid	C_21_H_9_O_13_^−^	469.0051	469.0049	0.43	470, 425, 407, 299	DT	[31]
14	2.768	Terflavin A	C_48_H_29_O_30_^−^	1085.0754	1085.074	0.46	933, 783, 631, 601, 451, 301	EE; DT	[31]
15	2.937	Punicalagin isomers	C_48_H_27_O_30_^−^	1083.0578	1083.059	−1.38	781, 601, 451, 301	EE; DT	[31,32]
16	3.131	HHDP—galloyl glucose isomer II	C_27_H_21_O_18_^−^	633.0725	633.0733	−1.26	301, 275	EE; DT	[31,32]
17	4.251	HHDP—galloyl glucose isomer III	C_27_H_21_O_18_^−^	633.0686	633.0733	−7.42	301, 275	EE; DT	[31,32]
18	4.639	HHDP—galloyl glucose isomer IV	C_27_H_21_O_18_^−^	633.0736	633.0733	0.47	301, 275	EE; DT	[31,32]
19	9.385	Trigaloyl hexoside	C_27_H_23_O_18_^−^	635.0895	635.0890	0.79	483, 465, 313, 301, 169, 125	EE; DT	[31]
20	9.385	Pterocarinin C	C_41_H_29_O_26_^−^	937.0968	937.0953	1.60	785, 635, 465, 301, 275, 169,125	DT	[33]
21	9.764	Tetragaloyl hexose	C_34_H_27_O_22_^−^	787.0977	787.0999	−2.79	635, 617, 465, 169	EE; DT	[34]
22	10.101	Ethyl gallate	C_9_H_9_O_5_^−^	197.0456	197.0455	0.51	169, 125	EE; DT	[31]
23	10.312	Pentagaloyl hexoside	C_41_H_31_O_26_^−^	939.1112	939.1109	0.32	787, 617, 465, 393, 241, 169	DT	[35]
24	10.481	Quercetin dihexoside	C_27_H_29_O_17_^−^	625.1412	625.1410	0.32	581, 579, 487, 463, 301, 300, 271, 169, 151	EE; DT	[36]
25	10.481	Isorhamnetin hexoside	C_22_H_21_O_12_^−^	477.1036	477.1038	−0.42	433, 314, 313, 301, 271, 169, 125	DT	[35,36]
26	10.565	Quercetin arabinoglycoside	C_26_H_27_O_16_^−^	595.1315	595.1305	1.68	300, 301, 271, 169	EE; DT	[37]
27	10.776	Quercetin galloyl hexoside	C_28_H_23_O_16_^−^	615.0982	615.0992	−1.62	463, 300, 301, 271	EE; DT	[38]
28	11.071	Quercetin hexoside	C_21_H_19_O_12_^−^	463.0882	463.0881	0.22	301, 300, 271	EE; DT	[38]
29	11.404	Galoyl quercetin	C_28_H_23_O_15_^−^	599.1040	599.1042	−0.33	463, 301, 285	EE; DT	[35,39]
30	11.362	Ellagic acid	C_14_H_5_O_8_^−^	300.9996	300.9984	3.99	284, 173, 145, 133	EE; DT	[32]
31	11.573	Hexoside kaempferol	C_21_H_19_O_11_^−^	447.0934	447.0933	0.22	285, 284, 255, 227	EE; DT	[32,35,40]
32	12.206	Quercetin	C_15_H_9_O_7_^−^	301.0370	301.0354	5.31	273, 169, 151, 134	EE; DT	[35]

Note: DT: dried tincture; EE: ethanolic extract/Rt: retention time; HHDP: hexahydroxydiphenol group, * [M − 2H]^2–^.

## Data Availability

Data are contained within the article and Supplementary Materials.

## Supplementary Materials

The following supporting information can be downloaded at: https://www.mdpi.com/article/10.3390/plants13152011/s1, Figure S1: (−)-MS of compounds **1** and **2** (*m/z* 181.0724 e 481.0635 [M − H]^−^) of Table 4. Figure S2: (−)-ESI-MSMS of compound **1** (*m/z* 181.0724 [M − H]^−^) of Table 4. Figure S3: (−)-ESI-MSMS of compound **2** (*m/z* 481.0635 [M − H]^−^) of Table 4. Figure S4: (−)-MS of compound **3** (mz 783.0685 [M − H]-) of Table 4. Figure S5: (−)-ESI-MSMS of compound **3** (*m/z* 783.0685 [M − H]^−^) of Table 4. Figure S6: (−)-MS of compound **4** (*m/z* 391.0303 [M−2H]^2–^) of Table 4. Figure S7: (−)-ESI-MSMS of compound **4** (*m/z* 391.0303 [M − 2H]^2–^) of Table 4. Figure S8: (−)-MS of compound **5** (*m/z* 387.1166 [M − H]^−^) of Table 4. Figure S9: (−)-ESI-MSMS of compound **5** (*m/z* 387.1166 [M − H]^−^) of Table 4. Figure S10: (−)-MS of compound **6** (*m/z* 781.0530 [M − H]^−^) of Table 4. Figure S11: (−)-ESI-MSMS of compound **6** (*m/z* 781.0530 [M − H]^−^) of Table 4. Figure S12: (−)-MS of compound **7** (*m/z* 633.0733 [M − H]^−^) of Table 4. Figure S13: (−)-ESI-MSMS of compound **7** (*m/z* 633.0733 [M − H]^−^) of Table 4. Figure S14: (−)-MS of compound **8** (*m/z* 541.0249 [M − 2H]^2−^) of Table 4. Figure S15: (−)-ESI-MSMS of compound **8** (*m/z* 541.0249 [M − 2H]^2−^) of Table 4. Figure S16: (−)-MS of compound **9** (*m/z* 331.0686 [M − H]^−^) of Table 4. Figure S17: (−)-ESI-MSMS of compound **9** (*m/z* 331.0686 [M − H]^−^) of Table 4. Figure S18: (−)-MS of compound **10** (*m/z* 951.0784 [M − H]^−^) of Table 4. Figure S19: (−)-ESI-MSMS of compound **10** (*m/z* 951.0784 [M − H]^−^) of Table 4. Figure S20: (−)-MS of compound **11** (*m/z* 933.0627 [M − H]^−^) of Table 4. Figure S21: (−)-ESI-MSMS of compound **11** (*m/z* 933.0627 [M − H]^−^) of Table 4. Figure S22: (−)-MS of compound **12** (*m/z* 169,0139 [M − H]^−^) of Table 4. Figure S23: (−)-ESI-MSMS of compound **12** (*m/z* 169.0139 [M − H]^−^) of Table 4. Figure S24: (−)-MS of compound **13** (*m/z* 469.0051 [M − H]^−^) of Table 4. Figure S25: (−)-ESI-MSMS of compound **13** (*m/z* 469.0051 [M − H]^−^) of Table 4. Figure S26: (−)-MS of compound **14** (*m/z* 1085.0754 [M − H]^−^) of Table 4. Figure S27: (−)-ESI-MSMS of compound **14** (*m/z* 1085,0754 [M − H]^−^) of Table 4. Figure S28: (−)-MS of compound **15** (*m/z* 1083.0578 [M − H]^−^) of Table 4. Figure S29: (−)-ESI-MSMS of compound **15** (*m/z* 1083.0578 [M − H]^−^) of Table 4. Figure S30: (−)-MS of compound **16** (*m/z* 633.0725 [M − H]^−^) of Table 4. Figure S31: (−)-ESI-MSMS of compound **16** (*m/z* 633.0725 [M − H]^−^) of Table 4. Figure S32: (−)-MS of compound **17** (*m/z* 633.0686 [M − H]^−^) of Table 4. Figure S33: (−)-ESI-MSMS of compound **17** (*m/z* 633.0686 [M − H]^−^). Figure S34: (−)-MS of compound **18** (*m/z* 633.0736 [M − H]^−^) of Table 4. Figure S35: (−)-ESI-MSMS of compound **18** (*m/z* 633.0736 [M − H]^−^) of Table 4. Figure S36: (−)-MS of compounds **19** and **20** (*m/z* 635.0895 e *m/z* 937.0968 [M − H]^−^) of Table 4. Figure S37: (−)-ESI-MSMS of compound **19** (*m/z* 635.0895 [M − H]^−^) of Table 4. Figure S38: (−)-ESI-MSMS of compound **20** (*m/z* 937.0968 [M − H]^−^) of Table 4. Figure S39: (−)-MS of compound **21** (*m/z* 787.0977 [M − H]^−^) of Table 4. Figure S40: (−)-ESI-MSMS of compound **21** (*m/z* 787.0977 [M − H]^−^) of Table 4. Figure S41: (−)-MS of compound **22** (*m/z* 197.0456 [M − H]^−^) of Table 4. Figure S42: (−)-ESI-MSMS of compound **22** (*m/z* 197.0456 [M − H]^−^) of Table 4. Figure S43: (−)-MS of compound **23** (*m/z* 939.1112 [M − H]^−^) of Table 4. Figure S44: (−)-ESI-MSMS of compound **23** (*m/z* 939.1112 [M − H]^−^) of Table 4. Figure S45: (−)-MS of compounds **24** and **25** (*m/z* 625.1412 e *m/z* 477.1036 [M − H]^−^) of Table 4. Figure S46: (−)-ESI-MSMS of compound **24** (*m/z* 625.1412 [M − H]^−^) of Table 4. Figure S47: (−)-ESI-MSMS of compound **25** (*m/z* 477.1036 [M − H]^−^) of Table 4. Figure S48: (−)-MS of compound **26** (*m/z* 595.1315 [M − H]^−^) of Table 4. Figure S49: (−)-ESI-MSMS of compound **26** (*m/z* 595.1315 [M − H]^−^) of Table 4. Figure S50: (−)-MS of compound **27** (*m/z* 615.0982 [M − H]^−^) of Table 4. Figure S51: (−)-ESI-MSMS of compound **27** (*m/z* 615.0982 [M − H]^−^) of Table 4. Figure S52: (−)-MS of compound **28** (*m/z* 463.0881 [M − H]^−^) of Table 4. Figure S53: (−)-ESI-MSMS of compound **28** (*m/z* 615.0982 [M − H]^−^) of Table 4. Figure S54: (−)-MS of compound **29** (*m/z* 599.1040 [M − H]^−^) of Table 4. Figure S55: (−)-ESI-MSMS of compound **29** (*m/z* 599.1040 [M − H]^−^) of Table 4. Figure S56: (−)-MS of compound **30** (*m/z* 301.0010 [M − H]^−^) of Table 4. Figure S57: (−)-ESI-MSMS of compound **30** (*m/z* 301.0010 [M − H]^−^) of Table 4. Figure S58: (−)-MS of compound **31** (*m/z* 447.0934 [M − H]^−^) of Table 4. Figure S59: (−)-ESI-MSMS of compound **31** (*m/z* 447.0934 [M − H]^−^) of Table 4. Figure S60: (−)-MS of compound **32** (*m/z* 301.370 [M − H]^−^) of Table 4. Figure S61: (−)-ESI-MSMS of compound **32** (*m/z* 301.0370 [M − H]^−^) of Table 4.
