# Supplementary material for: Cosmetic Preservative Potential and Chemical Composition of Lafoensia replicata Pohl. Leaves
Source: Plants (Basel). 2024 Jul 23;13(15):2011. doi: 10.3390/plants13152011 (PMC11314560; doi:10.3390/plants13152011)

SUPPLEMENTARY MATERIAL

Cosmetic preservative potential and chemical composition of *Lafoensia replicata* Pohl leaves

Débora Machado de Lima[1], Anna Lívia Oliveira Santos[2], Matheus Reis Santos de Melo[3], Denise Crispim Tavares[3], Carlos Henrique Gomes Martins[2], Raquel Maria Ferreira Sousa[1]*

[1]Institute of Chemistry, Federal University of Uberlândia, Av. João Naves de Ávila 2121, 38400-902, Uberlândia, MG, Brazil

[2]Institute of Biomedical Sciences, Federal University of Uberlândia, Av. João Naves de Ávila 2121, 38400-902, Uberlândia, MG, Brazil

[3]University of Franca, Av. Dr. Armando Salles Oliveira, 201, 14.404-600 Franca, São Paulo, Brazil

* Corresponding author: rsousa@ufu.br

Figure S1: (–)-MS of compounds 1 and 2 (*m/z* 181.0724 e 481.0635 [M - H]$^-$) of Table 4.

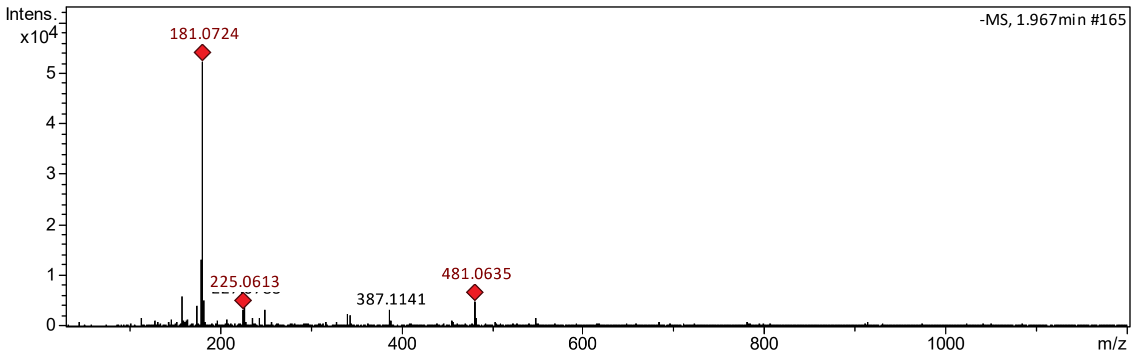

Figure S2: (–)-ESI-MS/MS of compound 1 (*m/z* 181.0724 [M - H]$^-$) of Table 4.

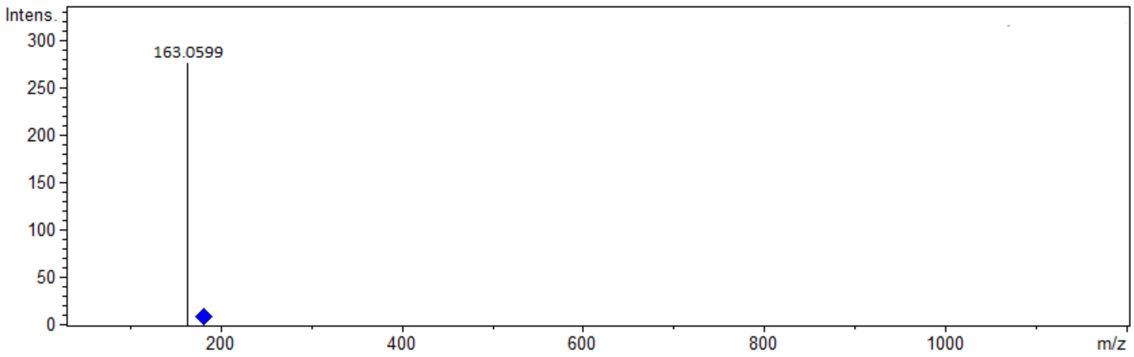

Figure S3: (–)-ESI-MS/MS of compound 2 (*m/z* 481.0635 [M - H]$^-$) of Table 4.

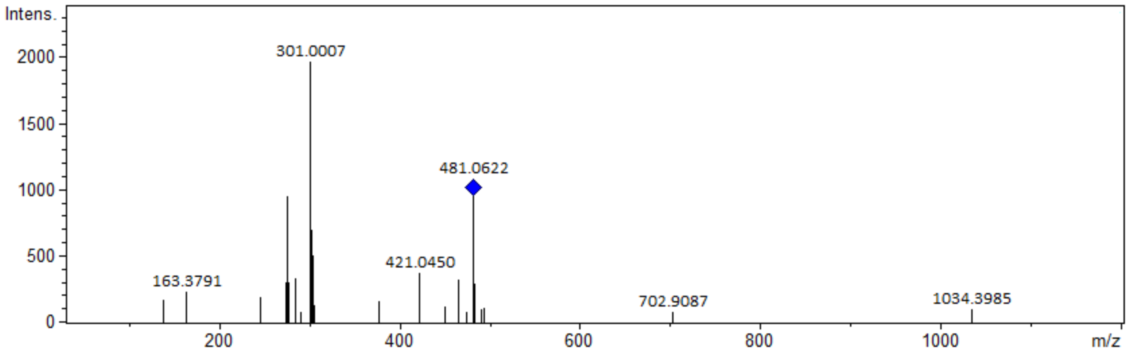

Figure S4: (–)-MS of compound 3 (m/z 783.0685 [M - H]-) of Table 4.

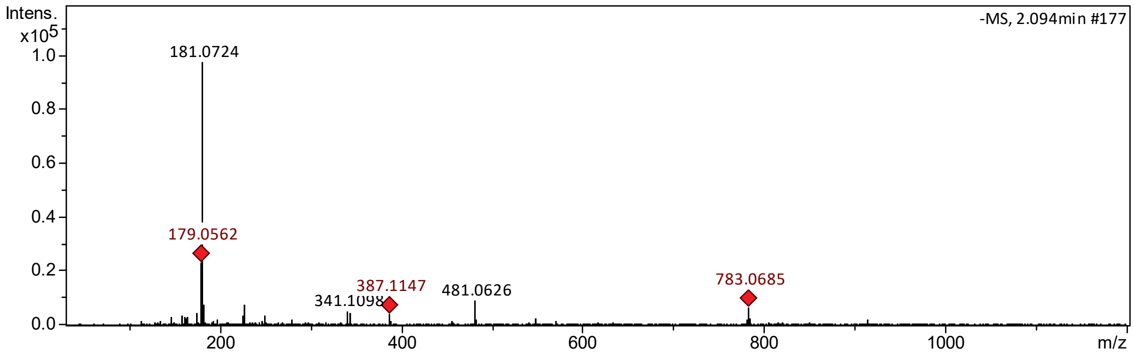

Figure S5: (–)-ESI-MS/MS of compound 3 (*m/z* 783.0685 [M - H]$^-$) of Table 4.

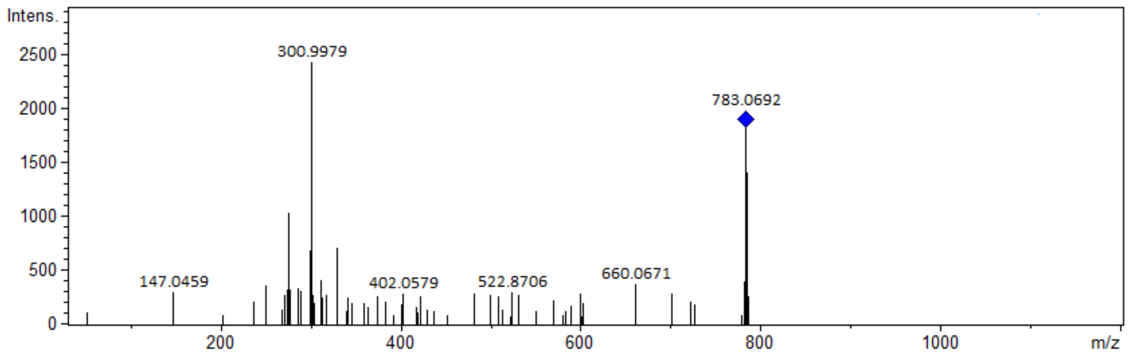

Figure S6: (–)-MS of compound 4 (*m/z* 391.0303 [M–2H]$^{2–}$) of Table 4.

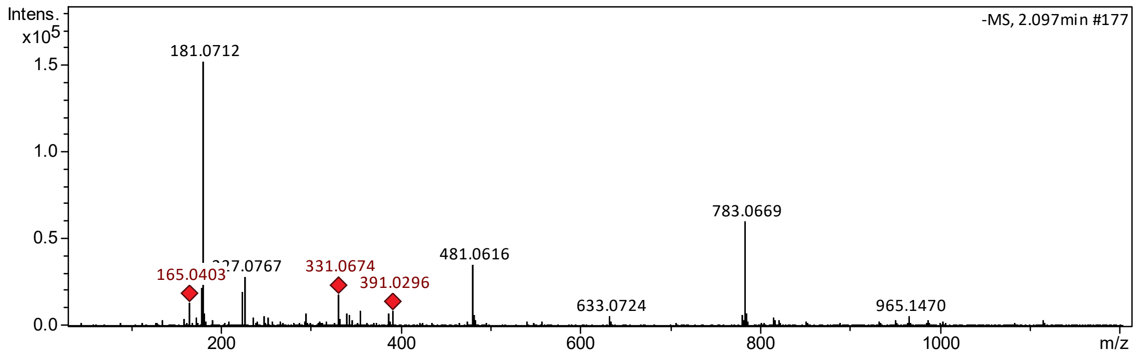

Figure S7: (–)-ESI-MS/MS of compound 4 (*m/z* 391.0303 [M–2H]$^{2–}$) of Table 4.

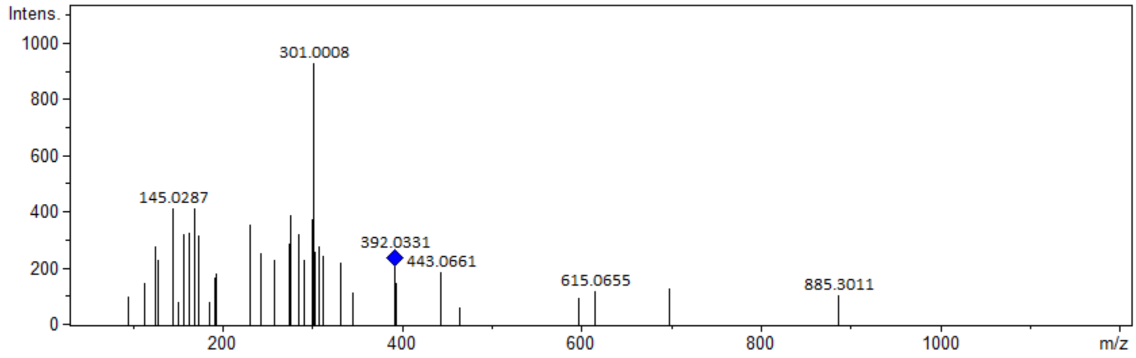

Figure S8: (–)-MS of compound 5 (*m/z* 387.1166 [M - H]$^-$) of Table 4.

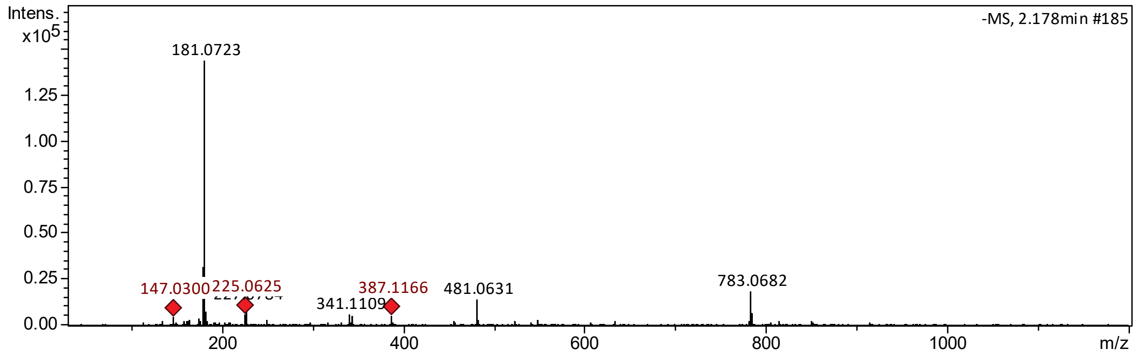

Figure S9: (–)-ESI-MS/MS of compound 5 (*m/z* 387.1166 [M - H]⁻) of Table 4.

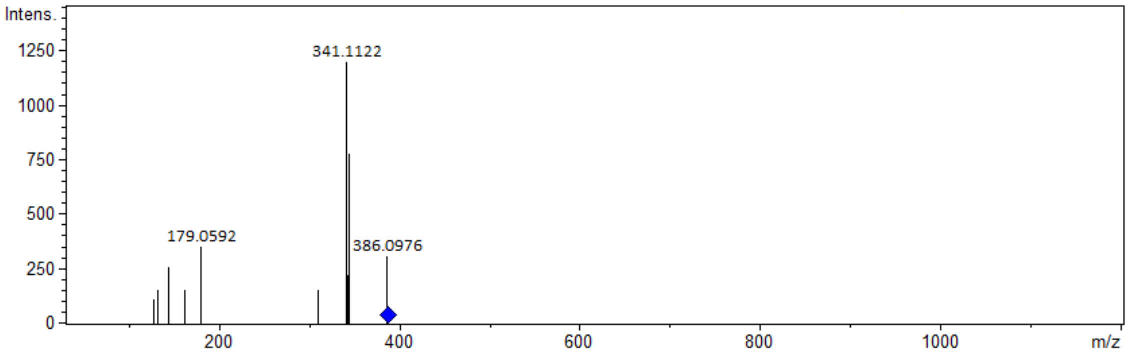

Figure S10: (–)-MS of compound 6 (*m/z* 781.0530 [M - H]⁻) of Table 4.

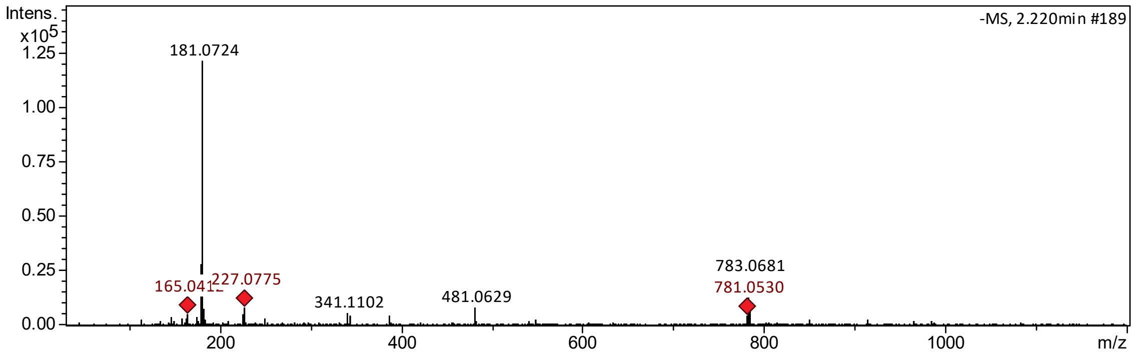

Figure S11: (–)-ESI-MS/MS of compound 6 (*m/z* 781.0530 [M - H]⁻) of Table 4.

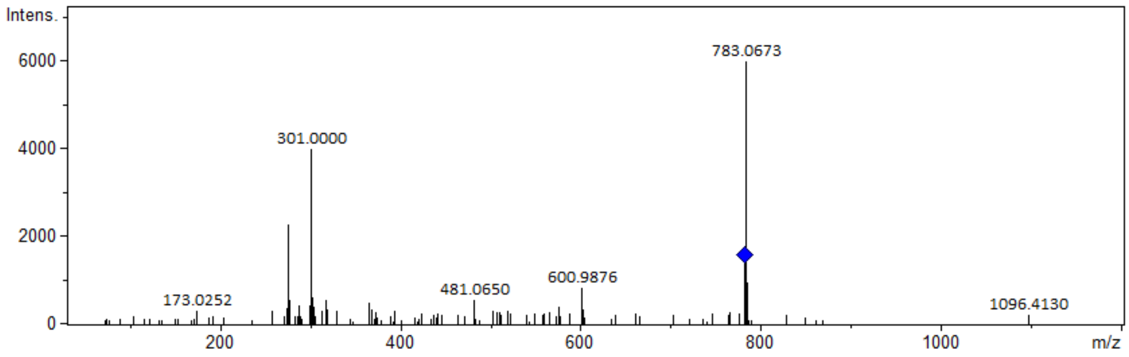

Figure S12: (–)-MS of compound 7 (*m/z* 633.0733 [M - H]⁻) of Table 4.

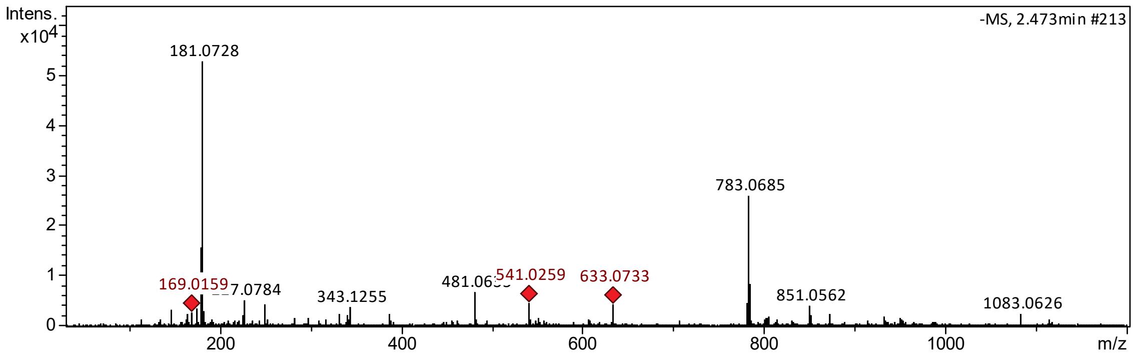

Figure S13: (–)-ESI-MS/MS of compound 7 (*m/z* 633.0733 [M - H]$^{-}$) of Table 4.

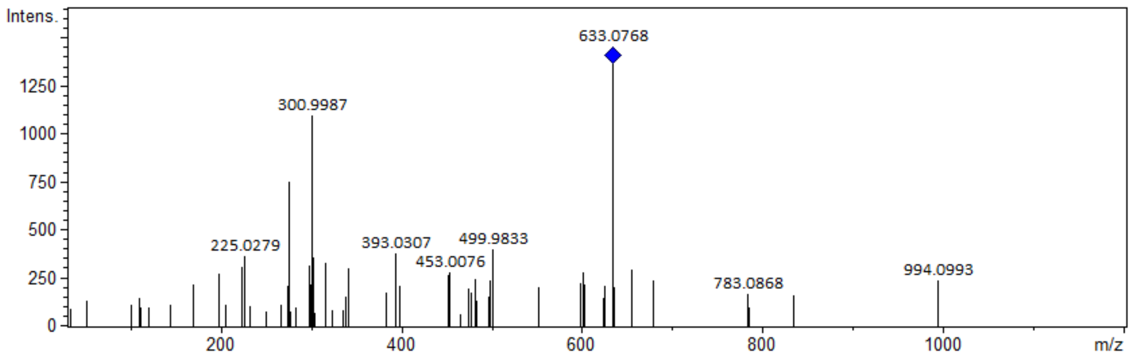

Figure S14: (–)-MS of compound 8 (*m/z* 541.0249 [M - 2H]$^{2-}$) of Table 4.

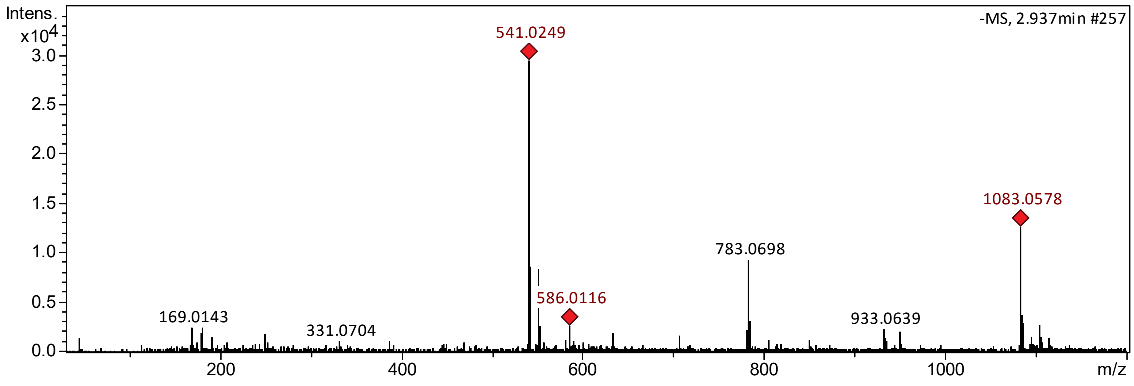

Figure S15: (–)-ESI-MS/MS of compound 8 (*m/z* 541.0249 [M - 2H]$^{2-}$) of Table 4.

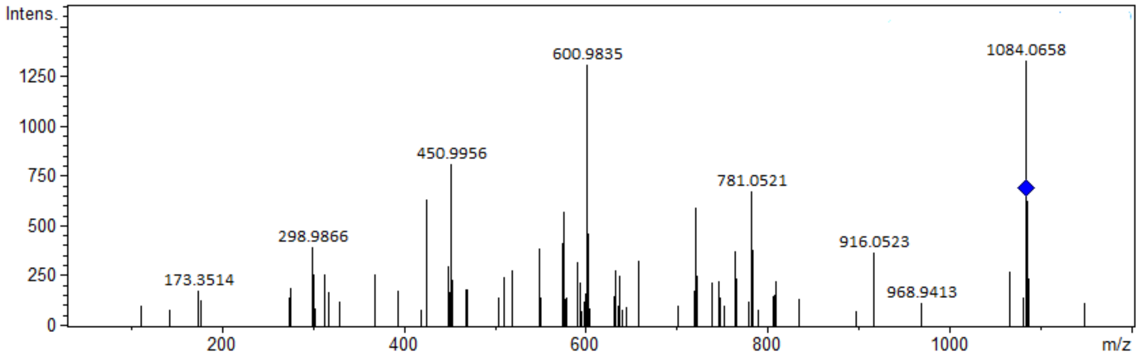

Figure S16: (–)-MS of compound 9 (*m/z* 331.0686 [M - H]$^{-}$) of Table 4.

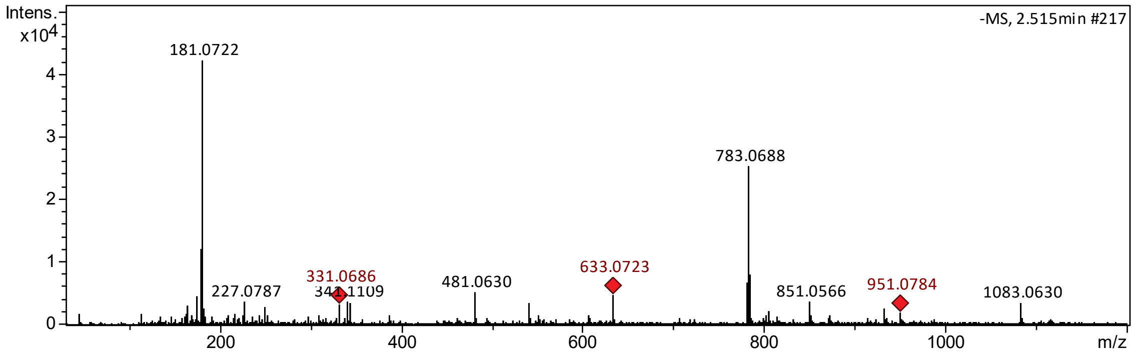

Figure S17: (–)-ESI-MS/MS of compound 9 (*m/z* 331.0686 $[M - H]^-$) of Table 4.

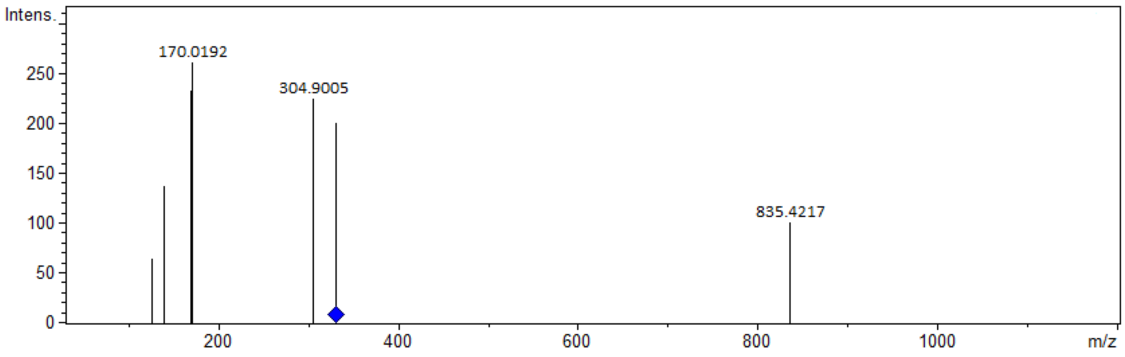

Figure S18: (–)-MS of compound 10 (*m/z* 951.0784 $[M - H]^-$) of Table 4.

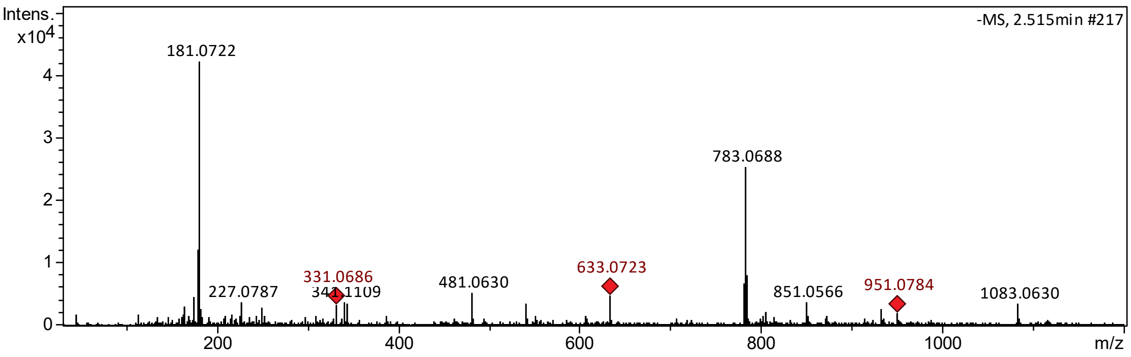

Figure S19: (–)-ESI-MS/MS of compound 10 (*m/z* 951.0784 $[M - H]^-$) of Table 4.

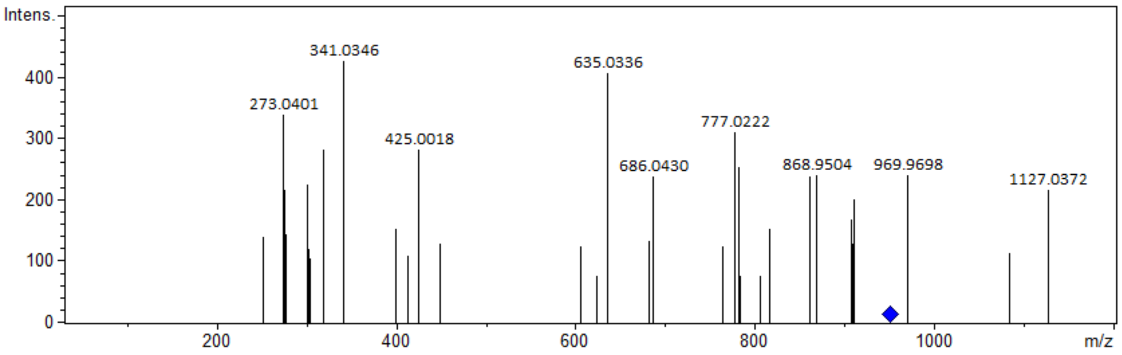

Figure S20: (–)-MS of compound 11 (*m/z* 933.0627 $[M - H]^-$) of Table 4.

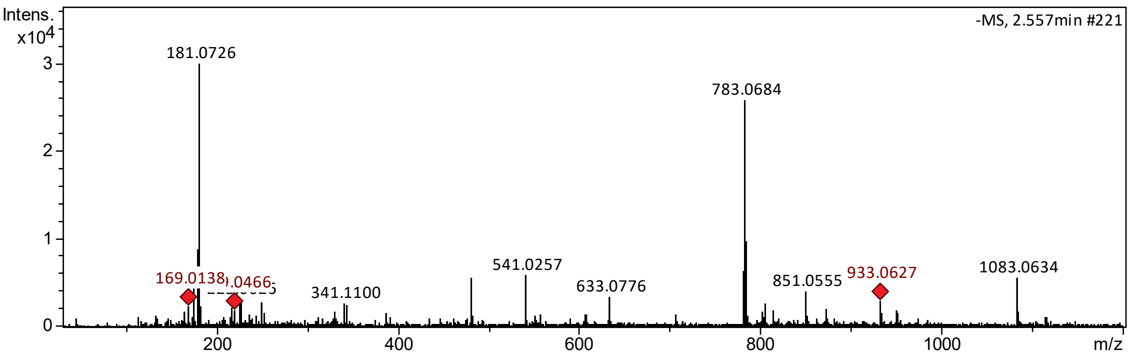

Figure S21: (–)-ESI-MS/MS of compound 11 (*m/z* 933.0627 $[M - H]^-$) of Table 4.

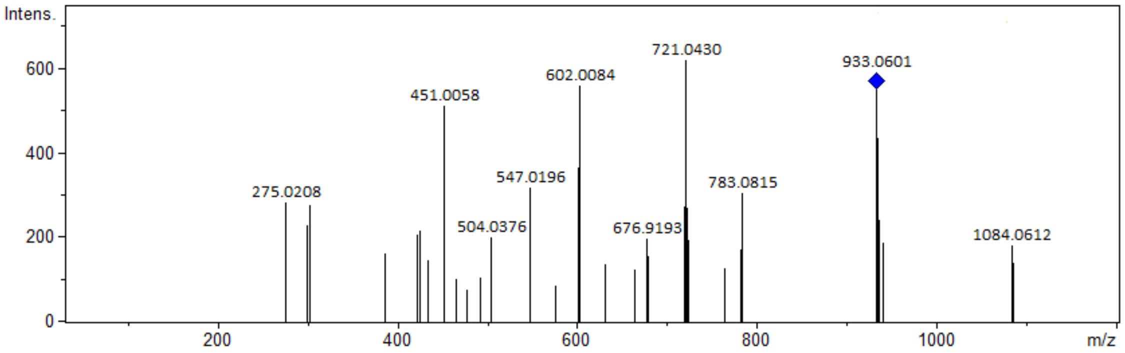

Figure S22: (–)-MS of compound 12 (*m/z* 169,0139 $[M - H]^-$) of Table 4.

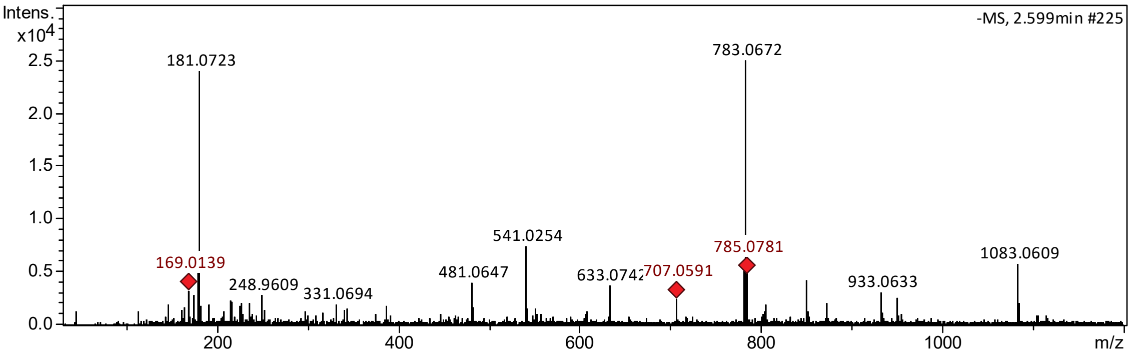

Figure S23: (–)-ESI-MS/MS of compound 12 (*m/z* 169.0139 $[M - H]^-$) of Table 4.

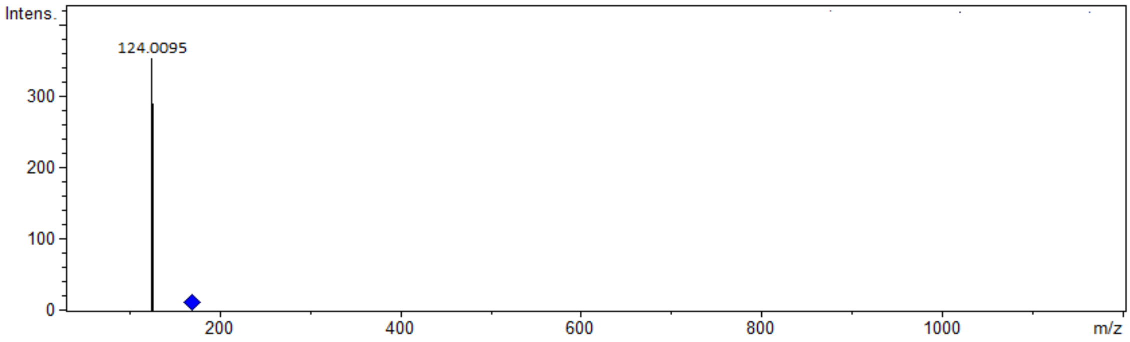

Figure S24: (–)-MS of compound 13 (*m/z* 469.0051 $[M - H]^-$) of Table 4.

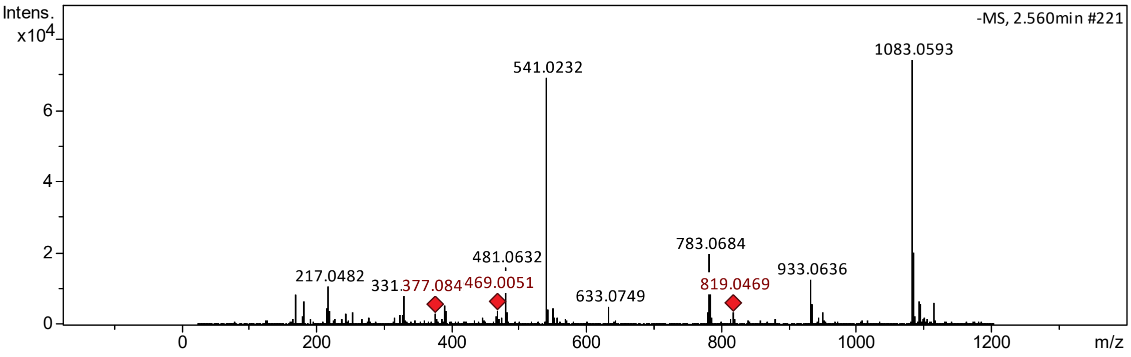

Figure S25: (–)-ESI-MS/MS of compound 13 (*m/z* 469.0051 [M - H]$^-$) of Table 4.

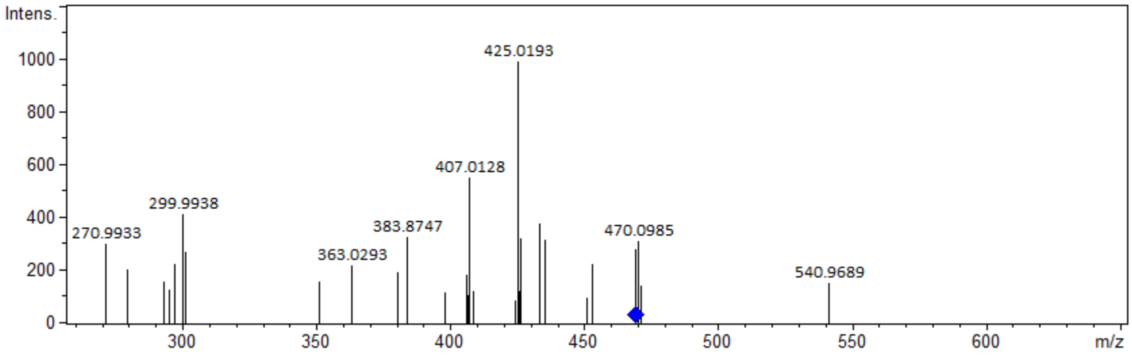

Figure S26: (–)-MS of compound 14 (*m/z* 1085.0754 [M - H]$^-$) of Table 4.

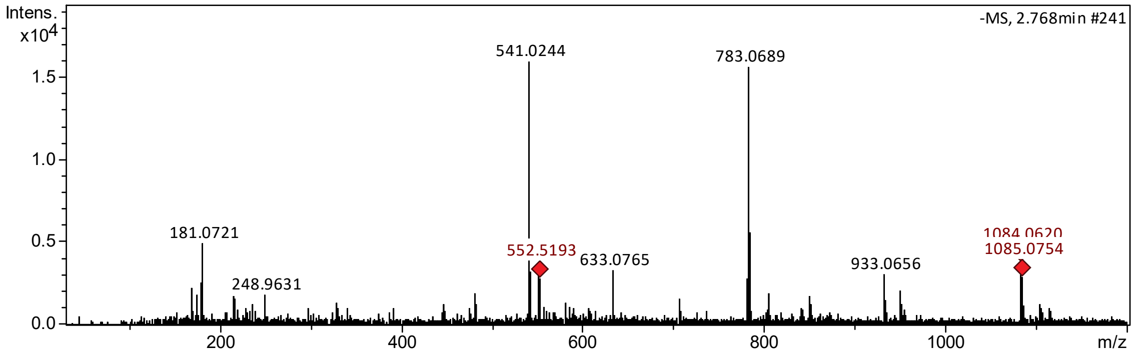

Figure S27: (–)-ESI-MS/MS of compound 14 (*m/z* 1085,0754 [M - H]$^-$) of Table 4.

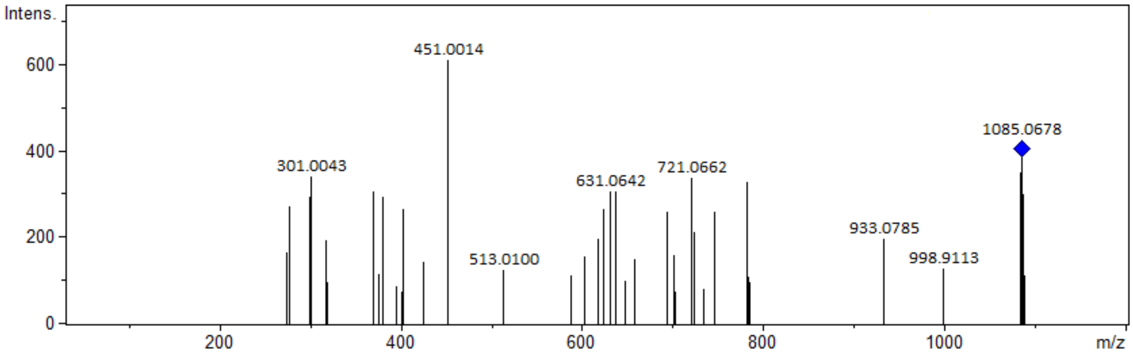

Figure S28: (–)-MS of compound 15 (*m/z* 1083.0578 [M - H]$^-$) of Table 4.

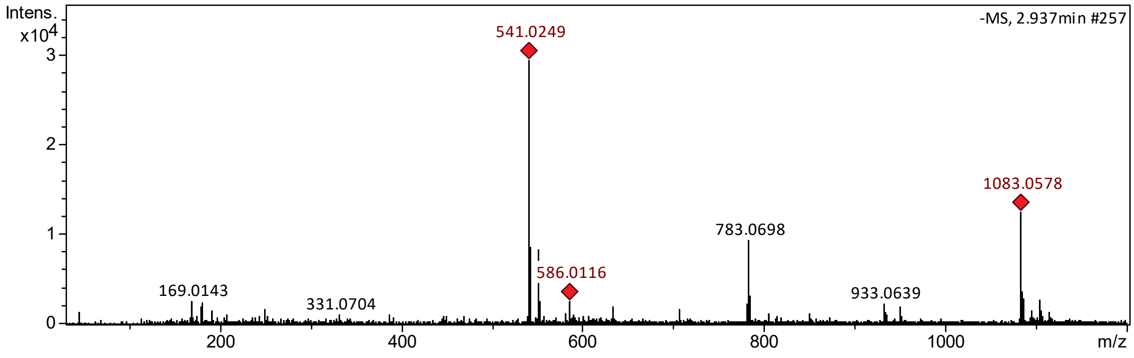

Figure S29: (–)-ESI-MS/MS of compound 15 (*m/z* 1083.0578 [M - H]⁻) of Table 4.

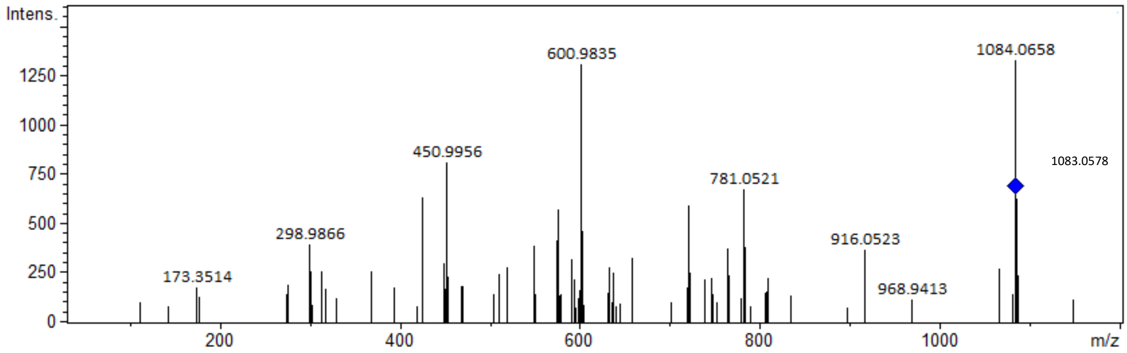

Figure S30: (–)-MS of compound 16 (*m/z* 633.0725 [M - H]⁻) of Table 4.

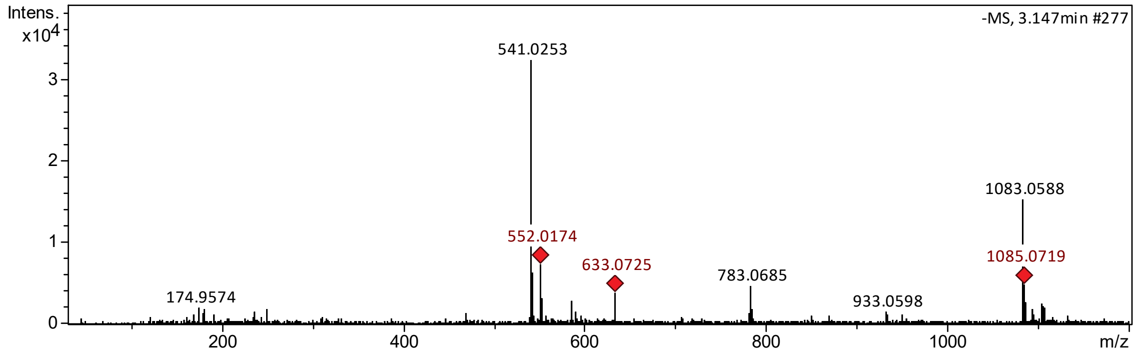

Figure S31: (–)-ESI-MS/MS of compound 16 (*m/z* 633.0725[M - H]⁻) of Table 4.

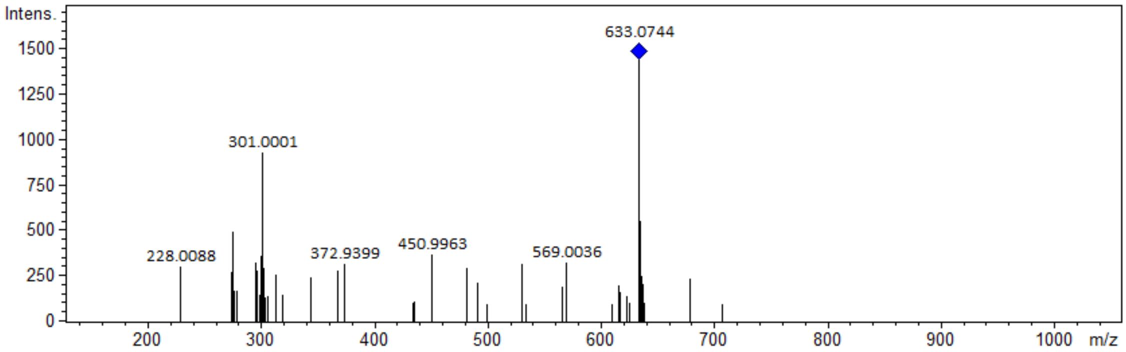

Figure S32: (–)-MS of compound 17 (*m/z* 633,0686 [M - H]⁻) of Table 4.

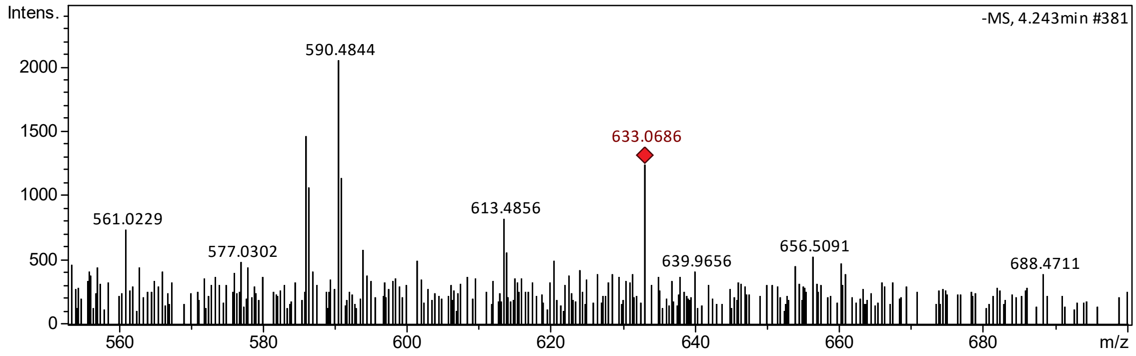

Figure S33: (–)-ESI-MS/MS of compound 17 (*m/z* 633.0686[M - H]$^-$).

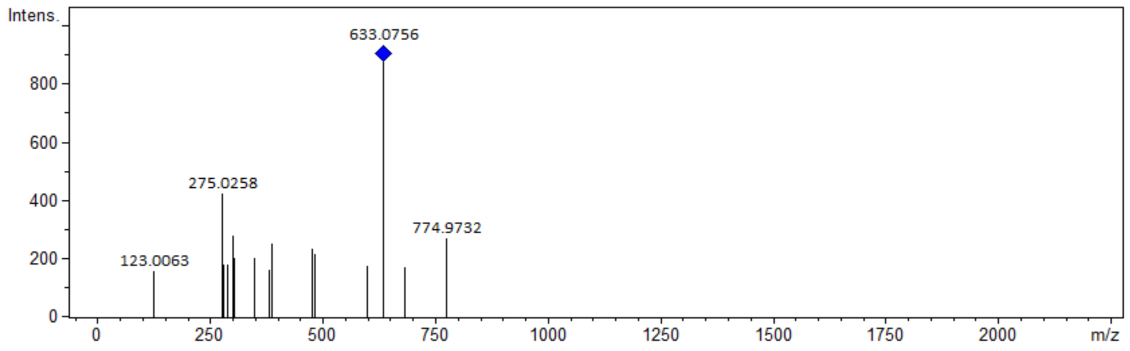

Figure S34: (–)-MS of compound 18 (*m/z* 633.0736 [M - H]$^-$) of Table 4.

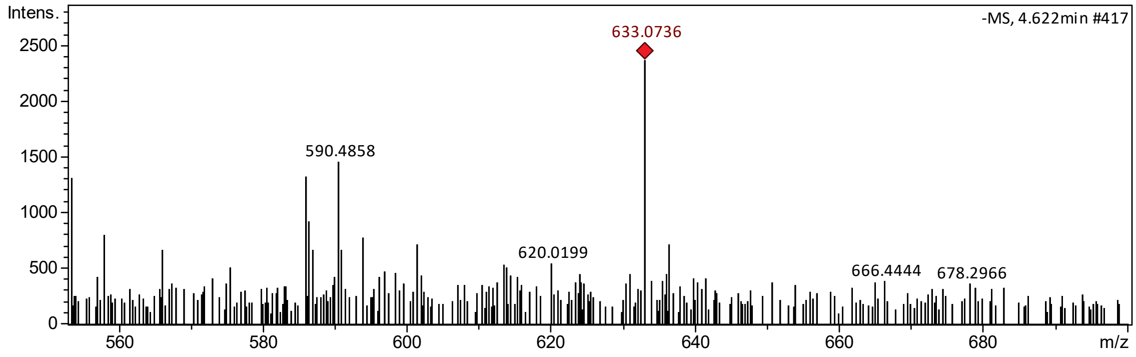

Figure S35: (–)-ESI-MS/MS of compound 18 (*m/z* 633.0736 [M - H]$^-$) of Table 4.

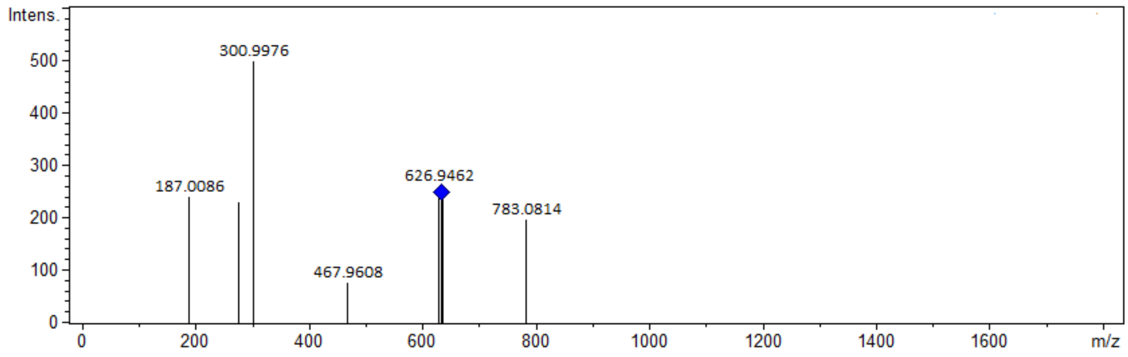

Figure S36: (–)-MS of compounds 19 and 20 (*m/z* 635.0895 e *m/z* 937.0968 [M - H]$^-$) of Table 4.

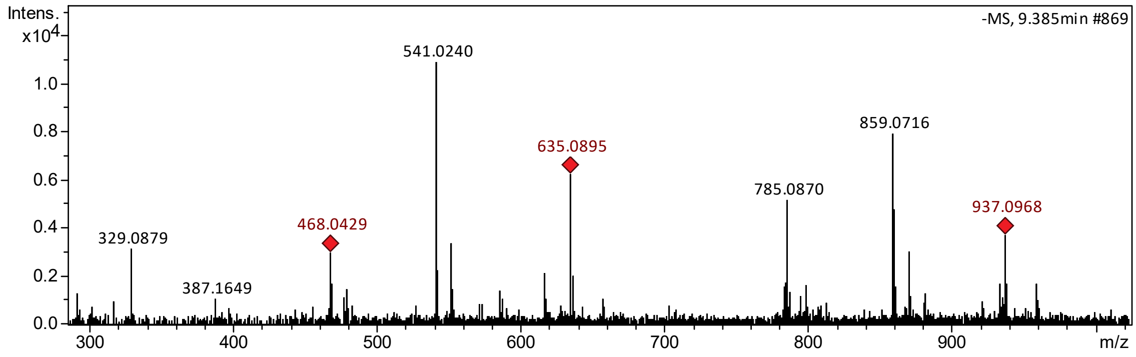

Figure S37: (–)-ESI-MS/MS of compound 19 (*m/z* 635.0895 [M - H]$^-$) of Table 4.

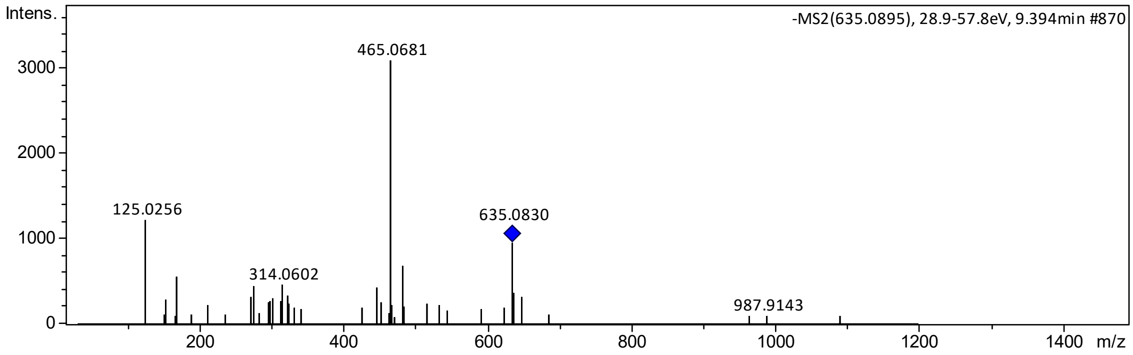

Figure S38: (–)-ESI-MS/MS of compound 20 (*m/z* 937.0968 [M - H]$^-$) of Table 4.

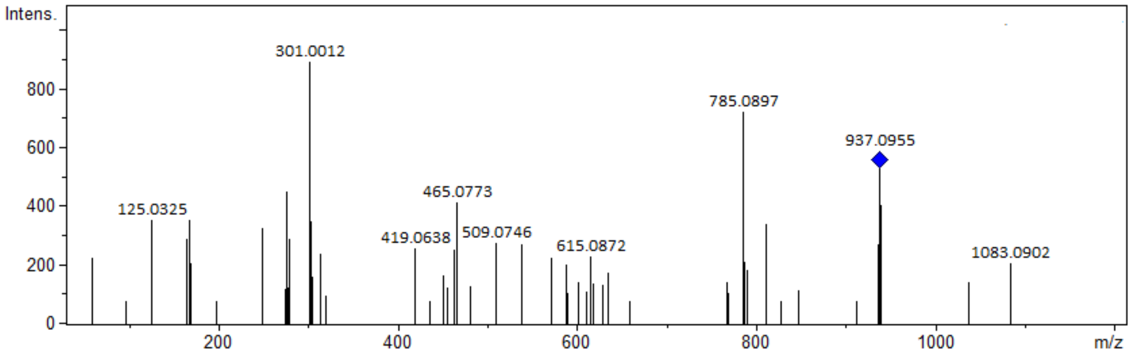

Figure S39: (–)-MS of compound 21 (*m/z* 787.0977 [M - H]$^-$) of Table 4.

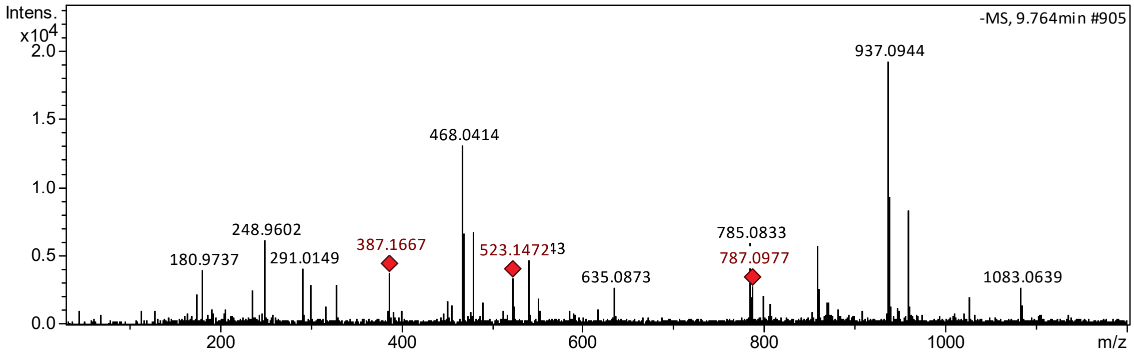

Figure S40: (–)-ESI-MS/MS of compound 21 (*m/z* 787.0977 [M - H]$^-$) of Table 4.

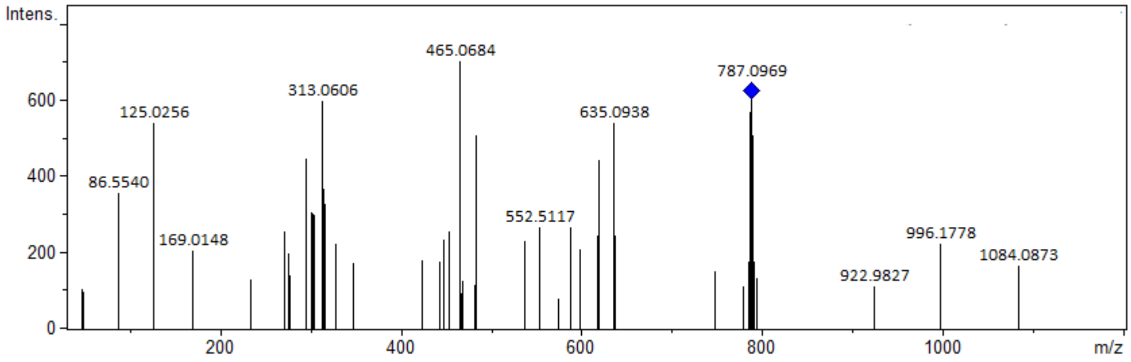

Figure S41: (–)-MS of compound 22 (*m/z* 197.0456 [M - H]$^-$) of Table 4.

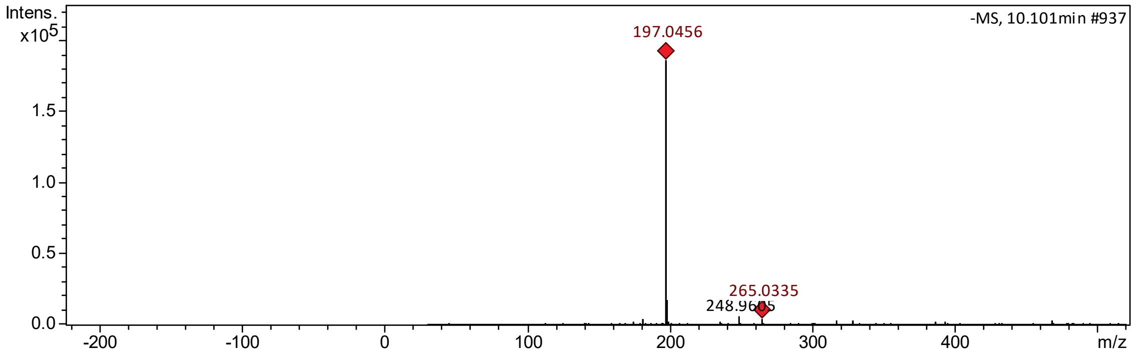

Figure S42: (–)-ESI-MS/MS of compound 22 (*m/z* 197.0456 [M - H]$^-$) of Table 4.

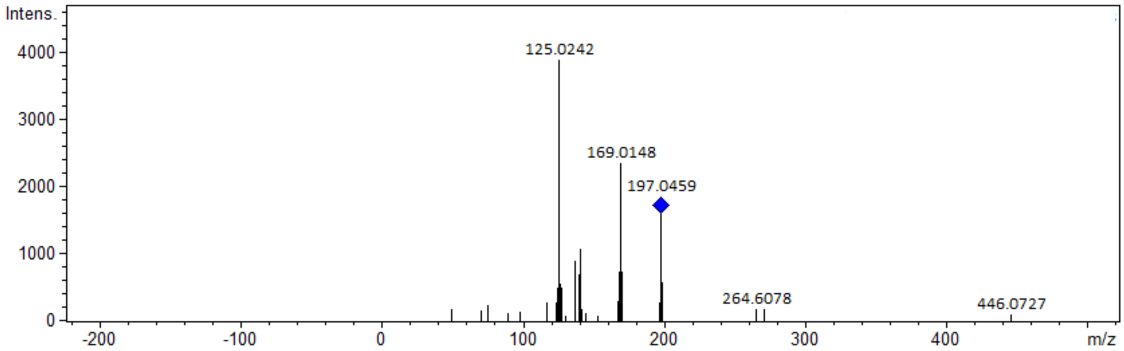

Figure S43: (–)-MS of compound 23 (*m/z* 939.1112 [M - H]$^-$) of Table 4.

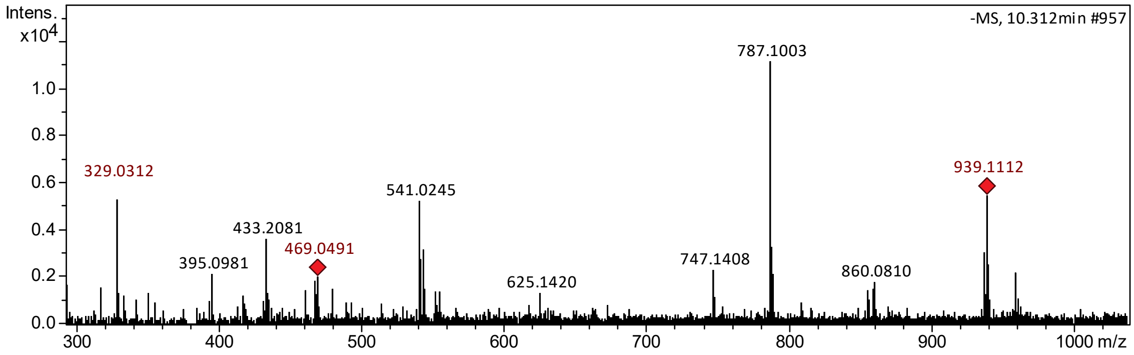

Figure S44: (–)-ESI-MS/MS of compound 23 (*m/z* 939.1112 [M - H]$^-$) of Table 4.

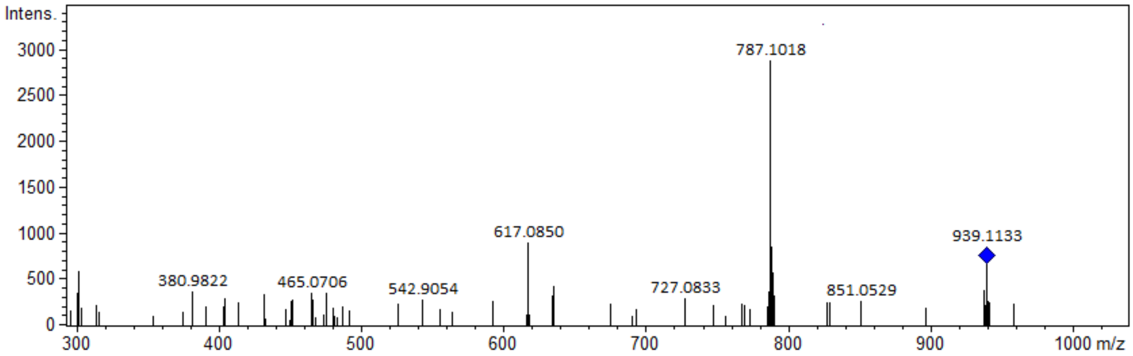

Figure S45: (–)-MS of compounds 24 and 25 (*m/z* 625.1412 e *m/z* 477.1036 [M - H]$^-$) of Table 4

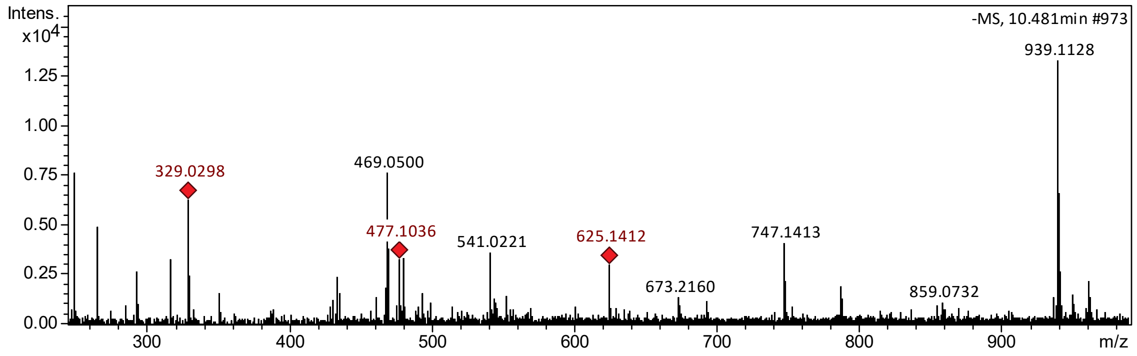

Figure S46: (–)-ESI-MS/MS of compound 24 (*m/z* 625.1412 [M - H]$^-$) of Table 4.

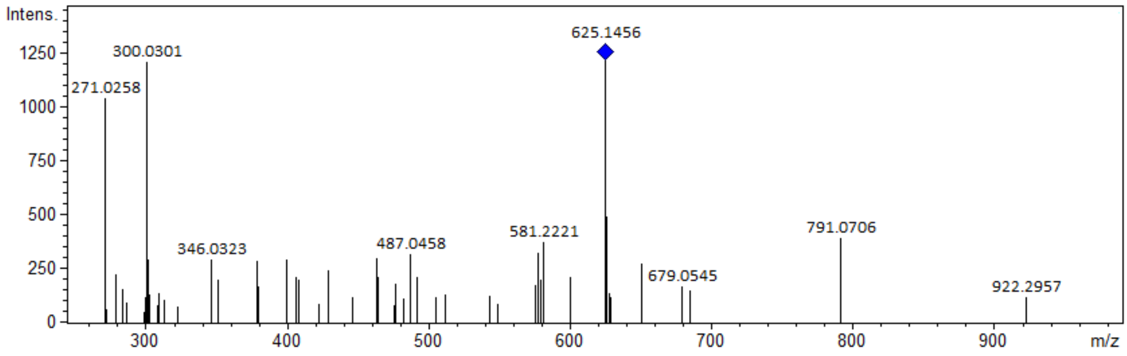

Figure S47: (–)-ESI-MS/MS of compound 25 (*m/z* 477.1036 [M - H]$^-$) of Table 4.

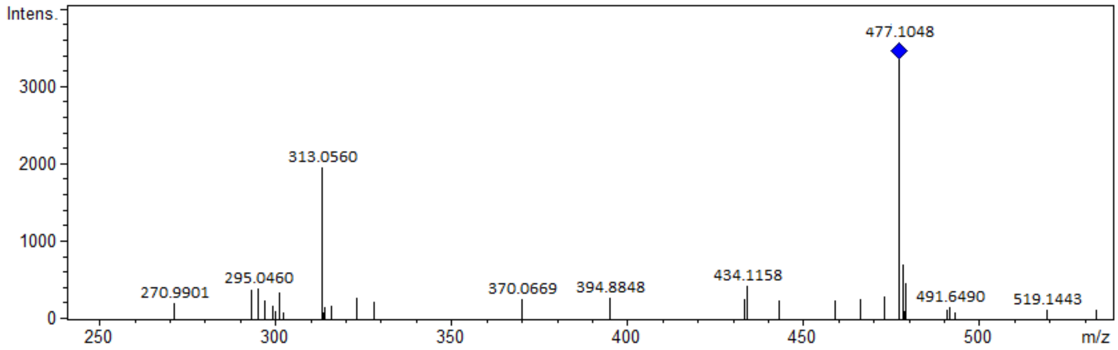

Figure S48: (–)-MS of compound 26 (*m/z* 595.1315 [M - H]$^-$) of Table 4.

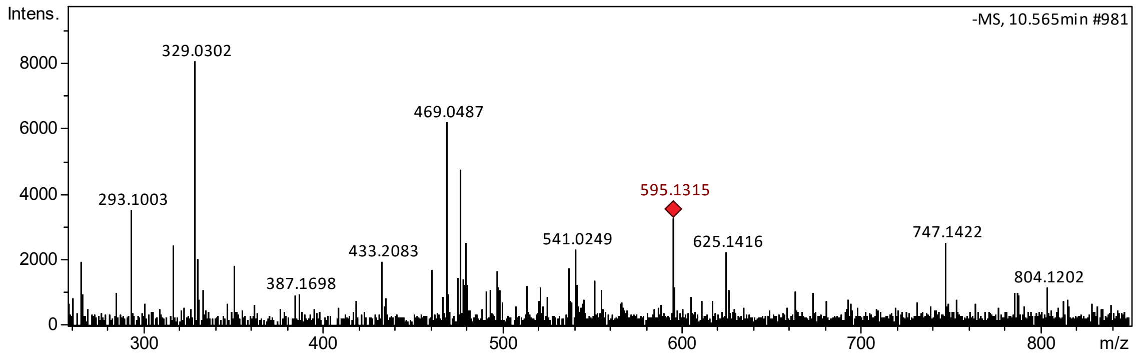

Figure S49: (–)-ESI-MS/MS of compound 26 (*m/z* 595.1315 [M - H]$^{-}$) of Table 4.

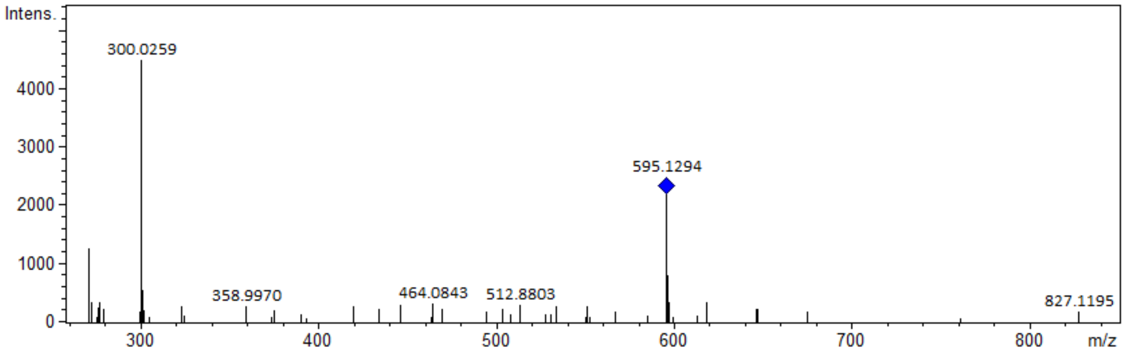

Figure S50: (–)-MS of compound 27 (*m/z* 615.0982 [M - H]$^{-}$) of Table 4.

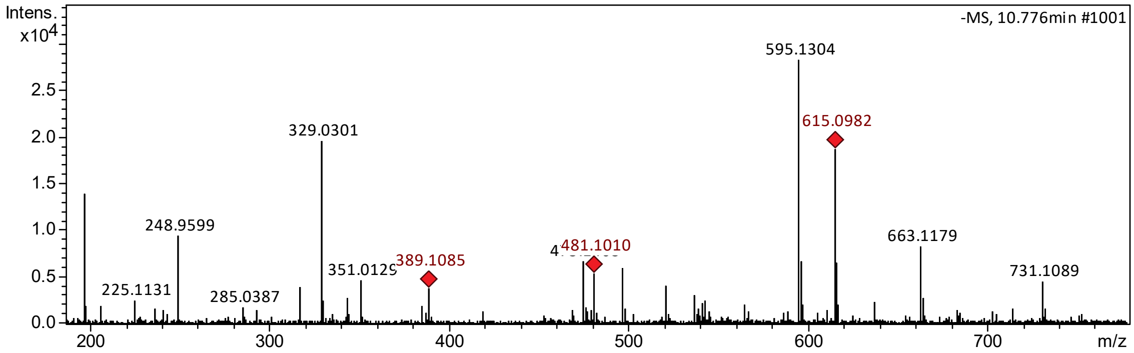

Figure S51: (–)-ESI-MS/MS of compound 27 (*m/z* 615.0982 [M - H]$^{-}$) of Table 4.

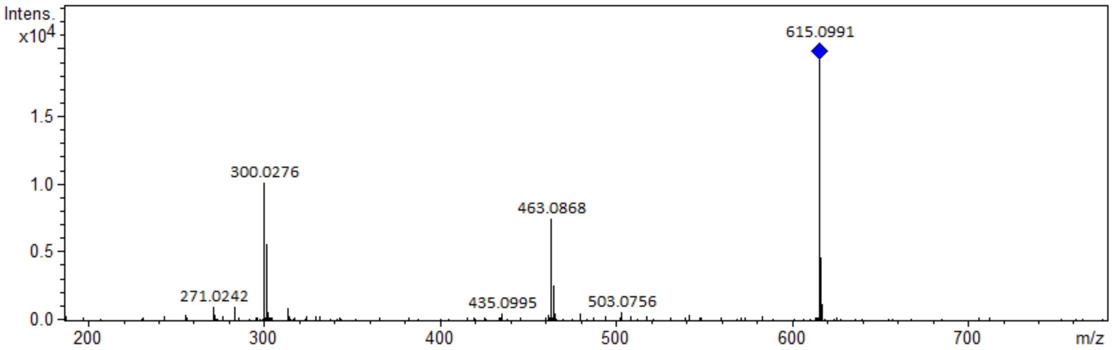

Figure S52: (–)-MS of compound 28 (*m/z* 463.0881 [M - H]$^{-}$) of Table 4.

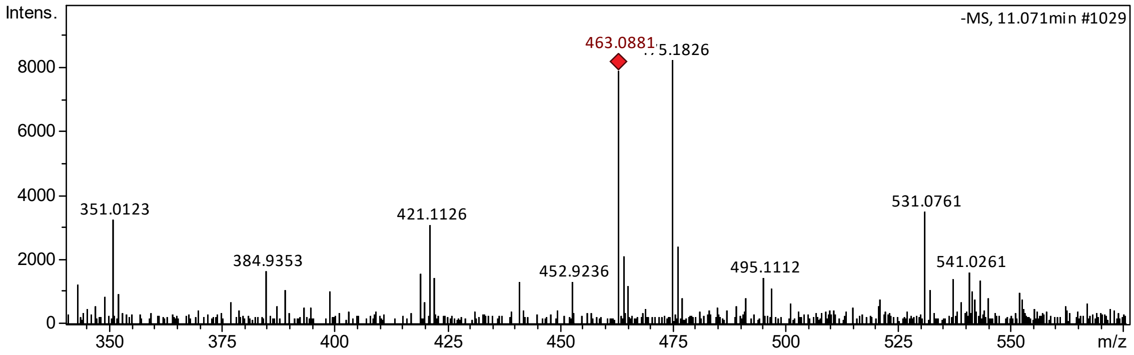

Figure S53: (–)-ESI-MS/MS of compound 28 (*m/z* 615.0982 [M - H]$^{-}$) of Table 4.

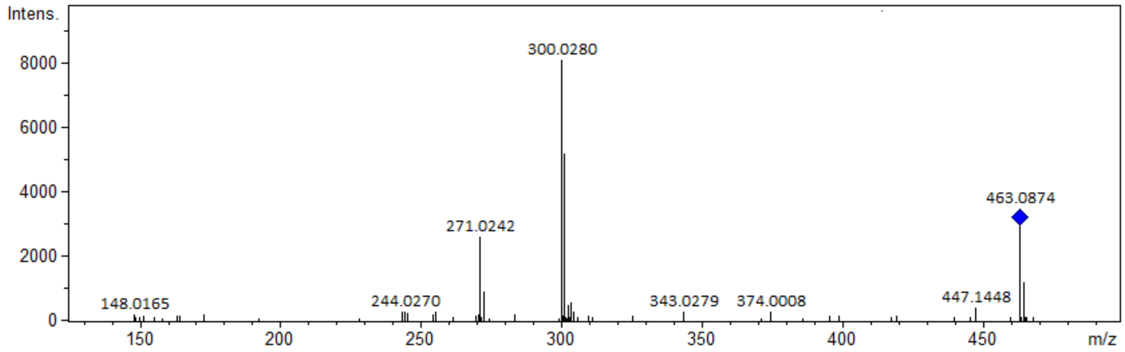

Figure S54: (–)-MS of compound 29 (*m/z* 599.1040 [M - H]$^{-}$) of Table 4.

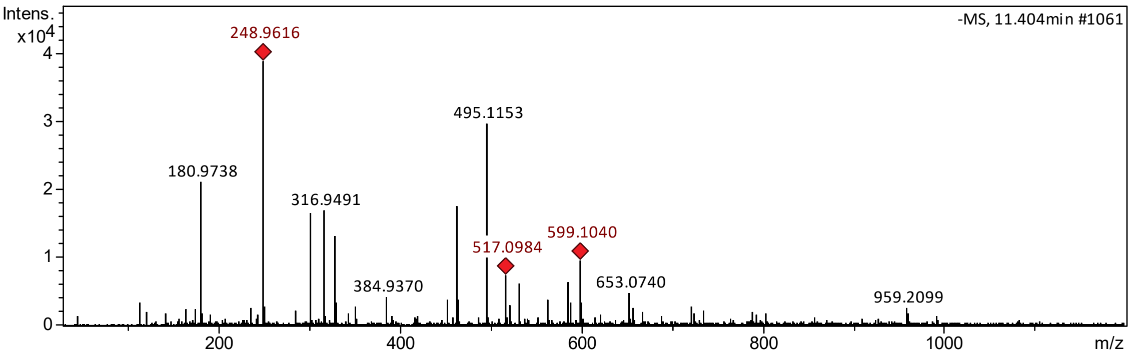

Figure S55: (–)-ESI-MS/MS of compound 29 (*m/z* 599.1040 [M - H]$^{-}$) of Table 4.

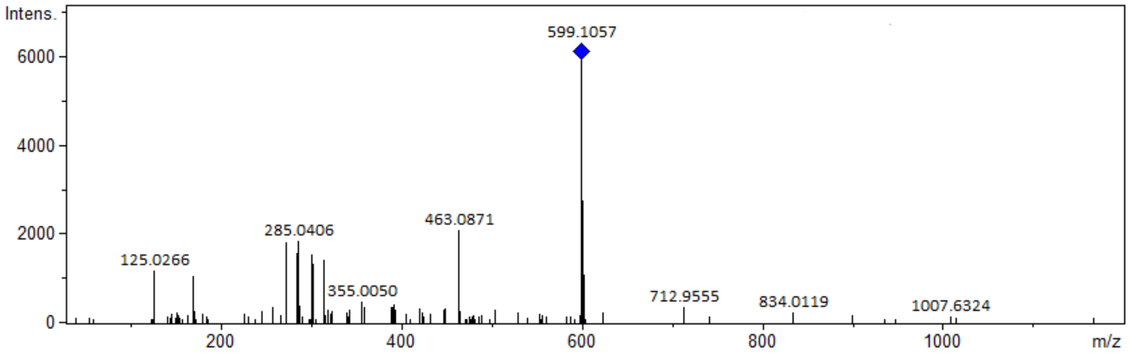

Figure S56: (–)-MS of compound 30 (*m/z* 301.0010 [M - H]$^{-}$) of Table 4.

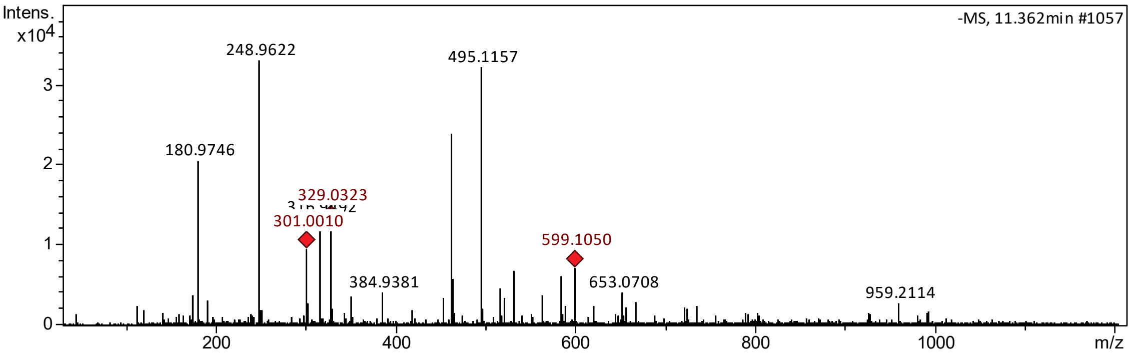

Figure S57: (–)-ESI-MS/MS of compound 30 (*m/z* 301.0010 $[M - H]^-$) of Table 4.

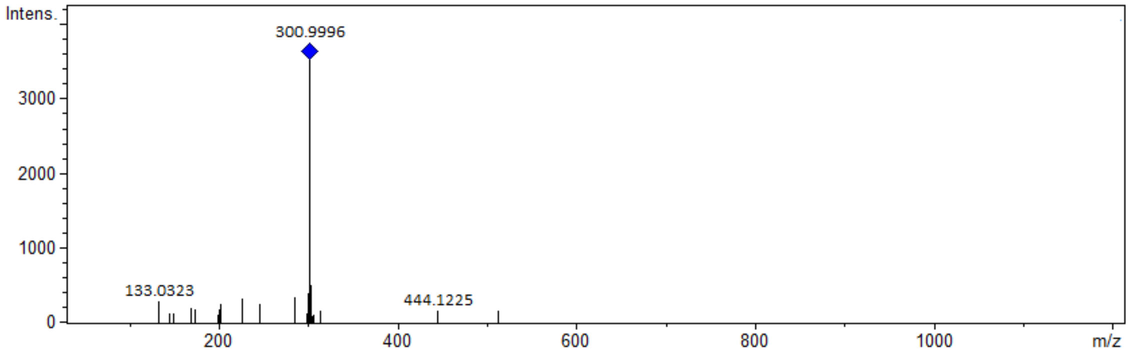

Figure S58: (–)-MS of compound 31 (*m/z* 447.0934 $[M - H]^-$) of Table 4.

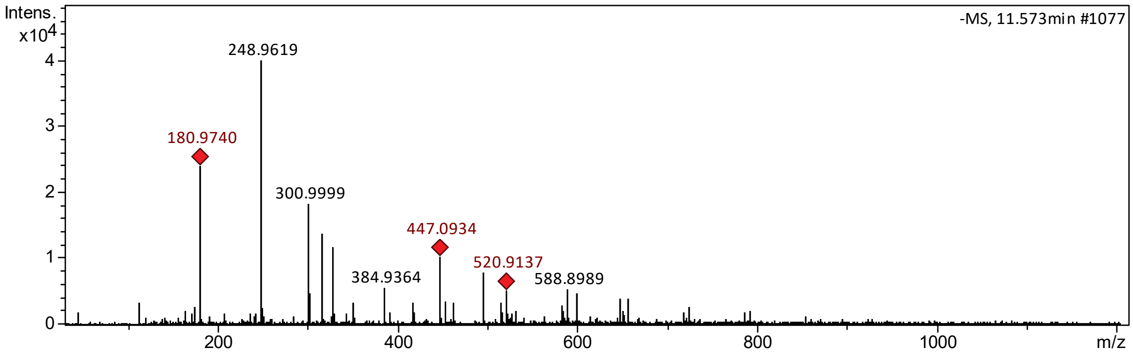

Figure S59: (–)-ESI-MS/MS of compound 31 (*m/z* 447.0934 $[M - H]^-$) of Table 4.

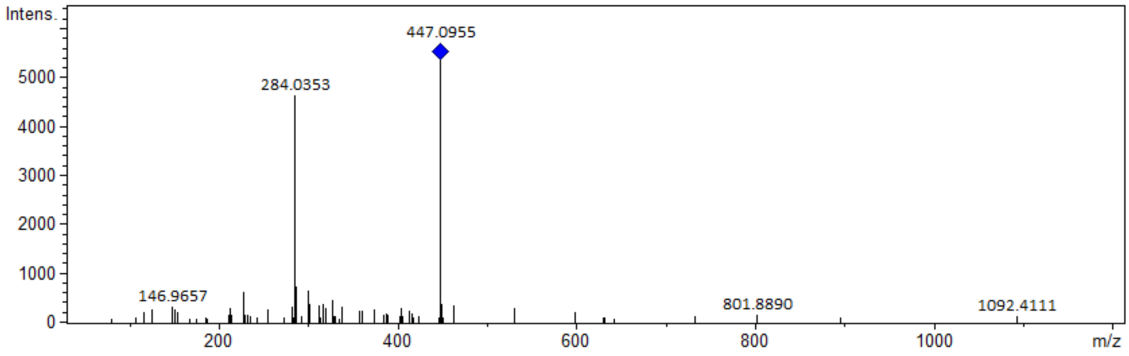

Figure S60: (–)-MS of compound 32 (*m/z* 301.370 $[M - H]^-$) of Table 4.

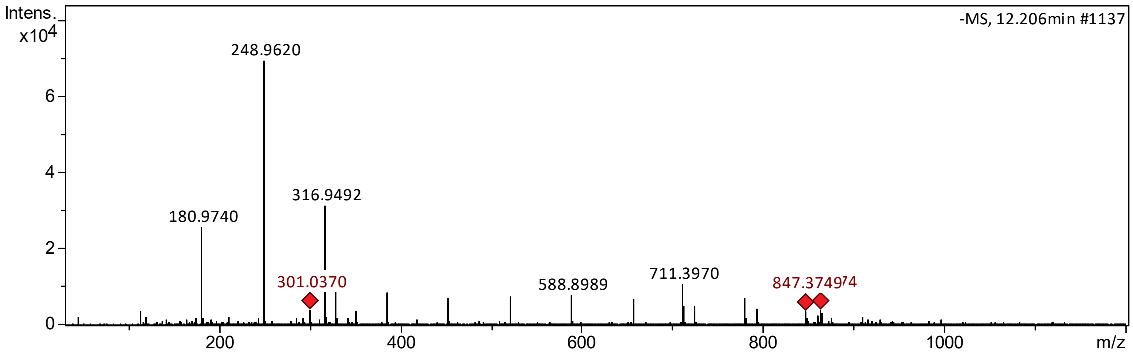

Figure S61: (–)-ESI-MS/MS of compound 32 (*m/z* 301.0370 [M - H]$^-$) of Table 4.

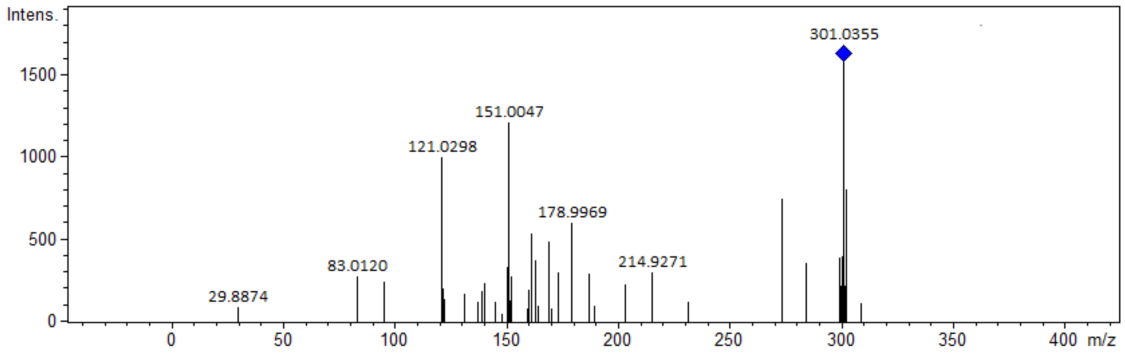

Supplement: Supplementary file 1 [file plants-13-02011-s001.zip › plants-3094249-supplementary.pdf]
